# Association of attenuated leptin signaling pathways with impaired cardiac function under prolonged high-altitude hypoxia

**DOI:** 10.1038/s41598-024-59559-6

**Published:** 2024-05-03

**Authors:** Jianan Wang, Shiying Liu, Lihong Sun, Zhanping Kong, Jiamin Chai, Jigang Wen, Xuan Tian, Nan Chen, Chengli Xu

**Affiliations:** 1 Institute of Basic Medical Sciences, School of Basic Medicine, Chinese Academy of Medical Sciences, Peking Union Medical College, Beijing, 100005 China; 2 Center for Experimental Animal Research, Institute of Basic Medical Sciences, Chinese Academy of Medical Sciences and Peking Union Medical College, Beijing, 100005 China; 3 Qinghai Provincial People’s Hospital, Xining, 810000 Qinghai China; 4 Center of Environmental and Health Sciences, Chinese Academy of Medical Sciences, Beijing, 100005 China

**Keywords:** Hypoxia, Antarctica, Humans, Rats, Leptin, Cardiovascular diseases, Cardiovascular diseases, Homeostasis

## Abstract

Cardiovascular function and adipose metabolism were markedly influenced under high altitudes. However, the interplay between adipokines and heart under hypoxia remains to be elucidated. We aim to explore alterations of adipokines and underlying mechanisms in regulating cardiac function under high altitudes. We investigated the cardiopulmonary function and five adipokines in Antarctic expeditioners at Kunlun Station (4,087 m) for 20 days and established rats exposed to hypobaric hypoxia (5,000 m), simulating Kunlun Station. Antarctic expeditioners exhibited elevated heart rate, blood pressure, systemic vascular resistance, and decreased cardiac pumping function. Plasma creatine phosphokinase-MB (CK-MB) and platelet-endothelial cell adhesion molecule-1 (sPecam-1) increased, and leptin, resistin, and lipocalin-2 decreased. Plasma leptin significantly correlated with altered cardiac function indicators. Additionally, hypoxic rats manifested impaired left ventricular systolic and diastolic function, elevated plasma CK-MB and sPecam-1, and decreased plasma leptin. Chronic hypoxia for 14 days led to increased myocyte hypertrophy, fibrosis, apoptosis, and mitochondrial dysfunction, coupled with reduced protein levels of leptin signaling pathways in myocardial tissues. Cardiac transcriptome analysis revealed leptin was associated with downregulated genes involved in rhythm, Na^+^/K^+^ transport, and cell skeleton. In conclusion, chronic hypoxia significantly reduced leptin signaling pathways in cardiac tissues along with significant pathological changes, thus highlighting the pivotal role of leptin in regulation of cardiac function under high altitudes.

## Introduction

More than one hundred million people worldwide reside at high altitudes above 2500 m. In individuals residing at high altitudes for entertainment, occupational, or military purposes, hypobaric hypoxia influences pulmonary circulation, blood cell volume, and cardiovascular function^1^. Chronic hypoxia leads to pulmonary vascular remodeling and pulmonary arterial hypertension (PAH), leading to increased right ventricular (RV) pressure load and potentially resulting in right heart failure^2^. Right ventricular hypertrophy and diastolic dysfunction contribute to a decrease in left ventricular mass^3,4^, end-diastolic volume (EDV), and stroke volume (SV)^5^, and impaired diastolic and contractile function^4,6^.

The heart, a high-energy-consuming organ, maintains the supply of oxygen and energy substrates throughout the body. Hypoxia significantly shifts heart-specific fuel rewiring with a greater reliance of the heart on mitochondrial glucose oxidation^7^. Fatty acids serve as the principal energy substrates for myocardial tissues, furnishing about 70% ATP for sustaining cardiac contractile function^8^.

Adipose tissues may affect the metabolism of cardiovascular system by secreting a wide range of bioactive factors, such as leptin, resistin, adiponectin and adipsin, thereby regulating cardiac metabolic health through both endocrine and paracrine ways^9,10^. Especially, the epicardial adipose tissue (EAT) shares a common microcirculation with the myocardium without barrier^11^. It directly furnishes energy to myocardial cells and has a closely dialogue with cardiovascular system through bioactive factors^12^. Leptin is sensitive to acute changes in energy intake and showed robust effects on energy expenditure, sympathetic nervous system activity, lipid metabolism, inflammation, and hypothalamic-pituitary function^13^. Adiponectin is believed to have anti-diabetic, anti-atherosclerotic, antioxidant, and anti-inflammatory effects^14^. Imbalance of adipose factors leptin, adiponectin, and resistin is associated with an increased risk of cardiovascular events, and inadequate signaling of adiponectin and leptin can lead to cardiovascular disease^15–17^. Extensive research has been conducted on the role of adipokines in the cardiovascular system, yielding complex and contradictory findings^16^.

In contrast to obesity-induced adipocyte hypertrophy, high-altitude hypoxia leads to reduced appetite and increased energy expenditure, resulting in weight loss and decreased fat mass, accelerating fat breakdown and inhibiting fat synthesis^18^. This suggests that the impact of hypoxia on fat metabolism factors may differ from obesity^13,19^. During the acute hypoxia exposure period (3–7 days), some studies reported an increase in blood leptin levels^20,21^, while another study observed none change in blood leptin levels^22^. Long-term residents at high altitudes showed a decreasing trend in leptin^23^. For example, high-altitude residents have significantly lower serum leptin concentrations compared to lowlanders, even after adjusting for age and body mass index^24^. Adult males chronic exposed to high altitudes showed a decrease in fasting serum leptin concentrations, which is related to a decrease in norepinephrine (NE) levels^25^. These results suggest that acute and chronic hypoxia may have different regulatory mechanisms for leptin.

The Kunlun Station, is located at the highest point in Dome A in Antarctica (80°22′01.6"S, 77°22′23.3"E, 4087 m), and is referred to as an "inaccessible extreme environment for humans"^26^. The extreme hypoxia and cold in Dome A may impact cardiopulmonary function and metabolism, providing unique evidence to clarify potential adipokines involved in the regulation of heart. This study aimed to investigate the key adipose factors related to cardiorespiratory function in expeditioners at the Antarctic Kunlun Station at 20th days. We further constructed a rat model exposed to simulated high altitudes (5000 m), equivalent to the oxygen partial pressure at Kunlun Station, to elucidate whether cardiac pathological changes under hypoxia associated with crucial metabolic factors. This study may provide a comprehensive understanding of the potential role of adipokines in regulating cardiovascular function under hypoxic conditions and may offer potential targets for the prevention and treatment of cardiovascular diseases at high altitudes.

## Results

### Significant alterations in cardiopulmonary function among expeditioners at kunlun station in antarctica

Compared to those of departure, expeditioners at Kunlun Station showed significantly decreased percutaneous arterial oxygen saturation (SpO_2_, Table 1, Fig. S1A, n = 23). Expeditioners at Kunlun Station exhibited significant decreases in forced vital capacity (FVC), vital capacity max (VC_MAX_), forced expiratory volume in 1 s (FEV_1_), and the ratio of forced expiratory flow at 50% to forced inspiratory flow at 50% (FEF_50_/FIF_50_), while significant increases in FEV_1_/FVC, FEV_1_/VC_MAX_, forced inspiratory volume in 1 s (FIV_1_)/FVC, FIV_1_/VC_MAX_, peak inspiratory flow (PIF), peak expiratory flow (PEF), maximum expiratory flow at 75% of forced vital capacity (MEF_75_), and maximal mid-expiratory flow at 75–25% of forced vital capacity (MMEF_75/25_; Table 1, Fig. S1A). The results indicated the lung ventilation function was still limited after 20 days of adaption at Kunlun Station.

**Table 1:** The cardiopulmonary function of Antarctic expeditioners (n = 23).

Characteristics	Departure	Kunlun Station	*P* value
Pulse oximetry			
SpO_2_ (%)	98.17 ± 0.39	84.00 ± 5.14	*P* < 0.0001
Pulmonary function			
VC_MAX_ (L)	5.06 ± 0.62	4.57 ± 0.55	*P* < 0.0001
FVC (L)	5.06 ± 0.63	4.52 ± 0.53	*P* < 0.0001
FEV_1_ (L)	4.17 ± 0.52	3.92 ± 0.47	*P* < 0.0001
FEV_1_/FVC (%)	82.79 ± 7.05	87.01 ± 6.32	*P* < 0.0001
FEV_1_/VC_MAX_ (%)	82.66 ± 7.16	85.99 ± 6.50	*P* < 0.0001
PEF (L/s)	10.77 ± 1.54	11.74 ± 1.46	*P* < 0.0001
PIF (L/s)	6.73 ± 2.00	8.29 ± 2.10	*P* < 0.0001
MEF_75_ (L/s)	8.54 ± 1.81	9.21 ± 2.11	*P* = 0.0004
MMEF_75/25_ (L/s)	4.22 ± 1.29	4.50 ± 1.50	*P* = 0.0184
FIV_1_/FVC (%)	92.78 ± 13.93	97.84 ± 4.87	*P* = 0.0039
FIV_1_/VC_MAX_ (%)	87.96 ± 13.54	96.47 ± 5.37	*P* < 0.0001
FEF_50_/FIF_50_ (%)	86.51 ± 39.88	70.08 ± 26.34	*P* = 0.0060
Cardiovascular function			
HR (bpm)	70.35 ± 8.98	85.87 ± 11.91	*P* < 0.0001
SBP (mmHg)	118.83 ± 10.36	126.78 ± 11.69	*P* = 0.0010
DBP (mmHg)	76.57 ± 9.42	85.48 ± 8.74	*P* < 0.0001
MAP (mmHg)	86.78 ± 9.29	95.96 ± 9.06	*P* < 0.0001
CO (L/min)	5.87 ± 0.83	4.97 ± 0.86	*P* = 0.0013
CI (L/min/m^2^)	3.20 ± 0.40	2.71 ± 0.35	*P* = 0.0011
SV (mL)	84.09 ± 11.56	58.96 ± 18.27	*P* < 0.0001
SI (mL/m^2^)	45.74 ± 5.78	31.91 ± 8.36	*P* < 0.0001
SVR (dyn·s·cm^−5^)	2049.22 ± 348.30	2700.00 ± 448.61	*P* < 0.0001
SVRI (dyn·s·cm^−5^·m^2^)	1118.22 ± 181.31	1481.83 ± 292.94	*P* = 0.0001
TFC (1/kΩ)	29.10 ± 3.68	26.23 ± 2.85	*P* = 0.0008
TFCI (1/kΩ/m^2^)	15.96 ± 2.65	14.36 ± 1.95	*P* = 0.0006
VI (1/1000/s)	58.43 ± 14.28	34.22 ± 10.17	*P* = 0.0003
ACI (1/100/s^2^)	103.87 ± 33.17	69.65 ± 21.42	*P* = 0.0003
STR (%)	0.34 ± 0.07	0.48 ± 0.12	*P* < 0.0001
PEP (ms)	102.96 ± 16.15	113.61 ± 19.62	*P* = 0.0145
LVET (ms)	299.83 ± 26.33	252.09 ± 39.18	*P* < 0.0001
Electrocardiogram			
HR (bpm)	70.00 ± 9.35	82.96 ± 11.04	*P* < 0.0001
QT interval (ms)	387.22 ± 21.82	375.22 ± 25.44	*P* = 0.0284
Corrected QT (ms)	415.48 ± 21.60	433.61 ± 24.05	*P* = 0.0035
P wave (ms)	106.52 ± 9.86	99.83 ± 11.41	*P* = 0.0279
RR interval (ms)	870.87 ± 113.39	736.43 ± 112.82	*P* = 0.0008
PP interval (ms)	871.00 ± 112.73	737.00 ± 112.46	*P* = 0.0003
P axis (deg)	59.57 ± 20.02	68.87 ± 16.93	*P* = 0.0121
QRS axis (deg)	66.22 ± 50.49	72.30 ± 56.82	*P* = 0.0111
Body composition			
Weight (kg)	70.53 ± 9.44	69.61 ± 8.10	*P* = 0.1138
Basic metabolism (Kcal)	1625.13 ± 133.45	1607.61 ± 114.88	*P* = 0.0460
BMI (Kg·m^−2^)	23.51 ± 3.34	23.18 ± 2.97	*P* = 0.0868

Data were expressed as Mean ± SD. *P*, compared to Departure.

*VC*_*MAX*_ vital capacity max, *FVC* Forced Vital Capacity, *FEV*_*1*_ Forced Expiratory Volume in 1 s, *PEF* peak expiratory flow, *PIF* peak inspiratory flow *MEF*_*75*_ maximum expiratory flow at 75% of forced vital capacity, *MMEF*_*75/25*_ maximal mid-expiratory flow at 75–25% of forced vital capacity, *FIV*_*1*_ forced inspiratory volume in 1 s, *FEF*_*50*_*/FIF*_*50*_ ratio of forced expiratory flow at 50% of forced vital capacity to forced inspiratory flow at 50% of forced vital capacity. *HR* heart rate, *SBP* systolic blood pressure, *DBP* diastolic blood pressure, *MAP* mean arterial pressure, *CO* Cardiac Output, *CI* cardiac index, *SV* stroke volume, *SI* stroke index, *SVR* systemic vascular resistance, *SVRI* systemic vascular resistance index, *TFC* thoracic fluid content, *TFCI* thoracic fluid content index, *VI* velocity index, *ACI* acceleration index, *PEP* pre-ejection period, *LVET* left ventricular ejection time, *STR* systolic time ratio, *BMI* body mass index.

Expeditioners at Kunlun Station also showed significant increases in heart rate (HR), systolic blood pressure (SBP), diastolic blood pressure (DBP), mean arterial pressure (MAP), systemic vascular resistance (SVR), systemic vascular resistance index (SVRI), pre-ejection period (PEP), and systolic time ratio (STR) in expeditioners at Kunlun Station (Table 1, Fig. S1B). Conversely, there were significant decreases in stroke volume (SV), stroke index (SI), cardiac output (CO), cardiac index (CI), thoracic fluid content (TFC), thoracic fluid content index (TFCI), acceleration index (ACI), velocity index (VI), and left ventricular ejection time (LVET; Table 1, Fig. S1B). In addition, electrocardiogram (ECG) recordings indicated a significant prolongation of corrected QT (QTc) interval (half of the expeditioners more than 440 ms) and a significant deviation in QRS axis and P axis at Kunlun Station (Table 1, Fig. S1C). The QT interval, RR interval, PP interval, and P wave were significantly shortener due to increased HR (Table 1, Fig. S1C). There were no statistically significant differences in body composition, except for a significant decrease in basic metabolism at the Kunlun Station (Table 1, Fig. S1D).

### Elevation of cardiovascular risk factors and decreased plasma leptin levels in antarctic expeditioners are associated with alterations in cardiopulmonary function

**Figure 1:**
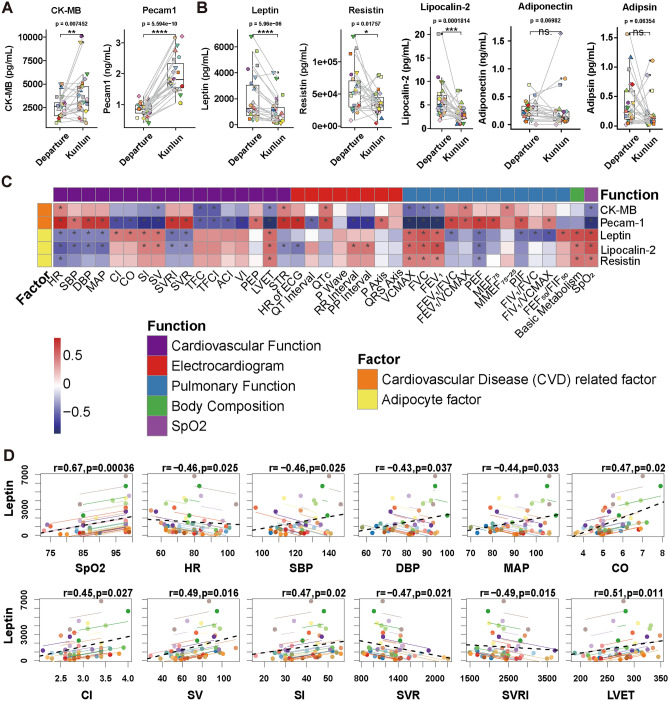
Alterations and correlation of cardiovascular risk factors and adipokines of the Kunlun Station expeditioners. (**A**) Plasma cardiovascular risk factors of expeditioners, including CK-MB and sPecam-1. (**B**) Plasma adipose/metabolic factor levels, including leptin, resistin, lipocalin-2, adiponectin, and adipsin of the expeditioners. (**C**) Heatmap shows correlations between plasma factors with altered indicators including cardiovascular function, cardiac conduction. (**D**) Correlation of plasma leptin with cardiovascular Indicators. n = 23. *p < 0.05 were considered significant (paired t-test or Wilcoxon signed-rank test). *p < 0.05 were considered significantly correlation (repeated measures correlation).

Compared to those at departure, plasma cardiovascular risk factors creatine phosphokinase-MB (CK-MB) and soluble platelet-endothelial cell adhesion molecule-1 (Pecam-1) were significantly increased at Kunlun Station (Fig. 1A). Leptin, resistin, and lipocalin-2 were significantly decreased at Kunlun Station (Fig. 1B). However, plasma adiponectin levels showed no statistically significant differences (Fig. 1B). Leptin has significant correlation with HR, SBP, DBP, MAP, CO, CI, SV, SI, SVR, SVRI, LVET, VC_MAX_, FVC, FEV_1_, FEV_1_/FVC, PEF, PIF, FIV_1_/FVC, FIV_1_/VC_MAX_, FEF_50_/FIF_50_, basic metabolism, and SpO_2_ (Fig. 1C,D), while lipocalin-2 has significant correlation with HR, SBP, DBP, MAP, SV, SI, SVR, SVRI, LVET, STR, VC_MAX_, FVC, FEV_1_, PEF, basic metabolism, and SpO_2_, and resistin has significant correlation with HR, LVET, VC_MAX_, FVC, FEV_1_, PEF, basic metabolism, and SpO_2_ (Fig. 1C). Due to the significant correlations between leptin and multiple indicators related to heart rate, blood pressure, peripheral vascular resistance, cardiac pumping and contractile function, and pulmonary ventilation function, leptin demonstrated the strongest correlation with cardiopulmonary function indicators.

### Simulation of 5000 m hypobaric hypoxic exposure induces pulmonary arterial hypertension, right ventricular hypertrophy, left ventricular contraction and diastolic changes in rats

To investigate the role of leptin in cardiovascular function, we established a targeted rat model exposed to a simulated 5000 m hypobaric hypoxic environment. SD rats were divided into groups exposed to hypoxia for 1 day (1d), 3 days (3d), 14 days (14d), and 28 days (28d) and corresponding control groups (Fig. S2A, n = 9). Hypoxic rats exhibited a sharp reduction in food intake and body weight despite having ad libitum access to food and water (Fig. S2B), body weight was significant lower in hypoxic groups than in normoxic groups (Fig. S2C,D). The heart wet weight/body weight (HW/BW) ratio significantly increased on 1d, 14d, and 28d of hypoxia exposure (Fig. S2E), which might be the result of losing in body weight under hypoxia. The ratio of heart wet weight to body length (nasoanal length) significantly increased on 14d of hypoxia exposure (Fig. S2F).

**Figure 2:**
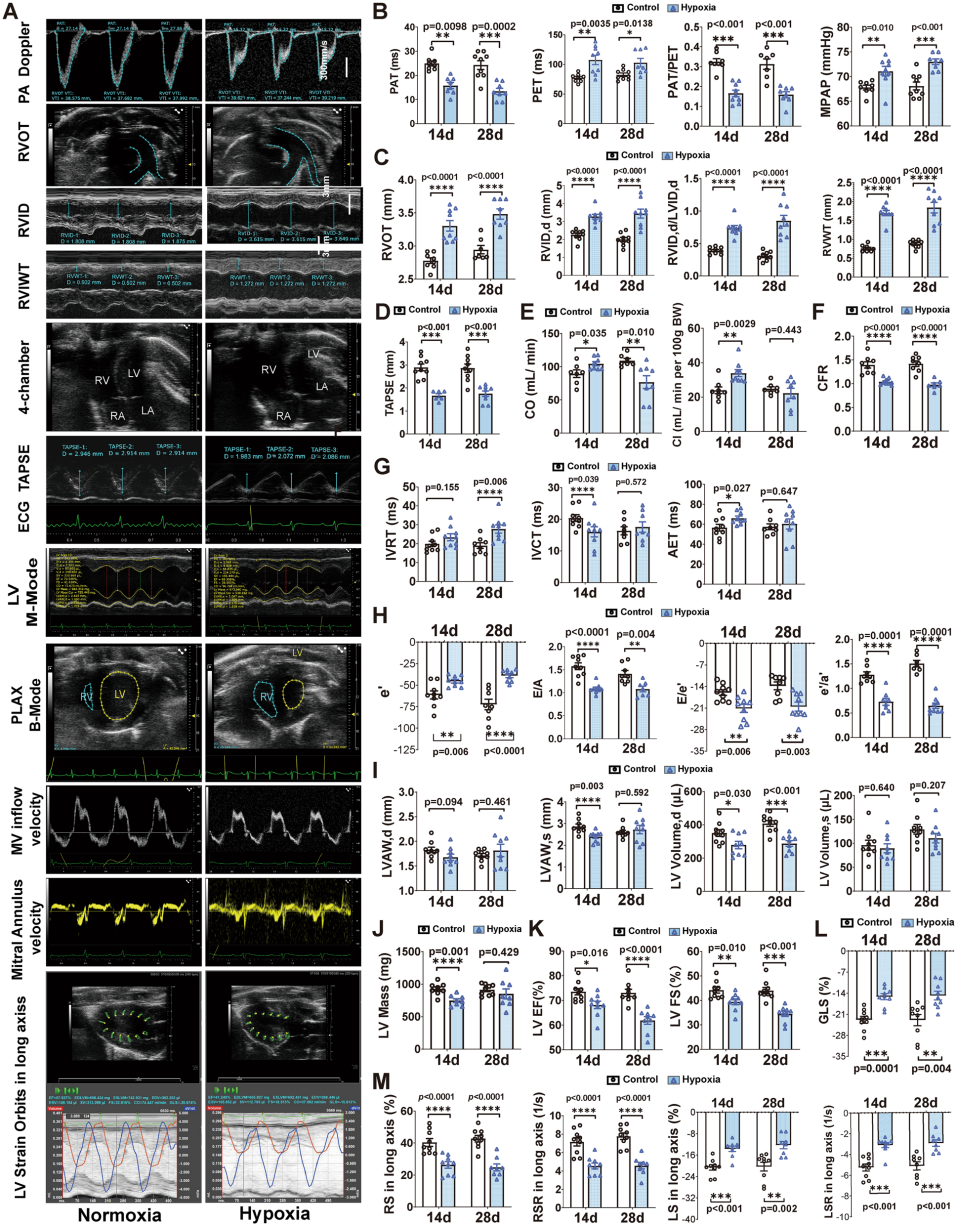
Hypoxia induced PVH, RVH, and impaired left ventricular contraction and relaxation in rats. (**A**) Representative images of transthoracic echocardiography in normoxic and hypoxic rats to quantify the indicators from (**B**–**M**). (**B**) Indicators of pulmonary artery hemodynamics. (**C**) Indicators of right ventricular function assessment. (**D**) Tricuspid annular plane systolic excursion (TAPSE). (**E**) Right ventricular cardiac output (CO) and cardiac index (CI). (**F**) Coronary flow reserve (CFR). (**G**) Indicators of left ventricular diastolic function, including left ventricular isovolumic relaxation time (IVRT), isovolumic contraction time (IVCT), aortic ejection time (AET). (**H**) Early diastolic mitral annular velocity (e'), ratio of mitral valve early diastolic velocity (MV E) to mitral valve atrial contraction velocity (MV A), E/e', and the ratio of e' to late diastolic mitral annular velocity (e'/a'). (**I**) Left ventricular anterior wall thickness at end-diastole (LVAW, d) and at end-systole (LVAW,s) , left ventricular volume at end-diastole (LV Volume, d), and left ventricular volume at end- systole (LV Volume, s). (**J**) LV mass. (**K**) Left ventricular ejection fraction (EF) and fractional shortening (FS). (**L**) Left ventricular global longitudinal strain (GLS). (**M**) Quantification of radial strain (RS), radial strain rate (RSR), longitudinal strain (LS), longitudinal strain rate (LSR). n = 7–9. Results are presented as mean ± SEM. The *p* values are shown on the top of compared group by unpaired t-test.

Echocardiography revealed hypoxia induced PAH, right ventricular hypertrophy (RVH), and left ventricular systolic and diastolic changes (Fig. 2A, n = 7 ~ 9). Hypoxia-induced PAH was suggested by a significant increase in pulmonary artery acceleration time (PAT) and a significant decrease in pulmonary artery ejection time (PET), as well as a significant increase in PAT/PET, mean pulmonary arterial pressure (MPAP; Fig. 2A,B), and the right ventricular outflow tract (RVOT, Fig. 2A,C) in hypoxic rats. Ultrasound examination of the right heart revealed significant increases in the right ventricular internal diameter at end-diastole (RVID,d), the ratio of RVID,d to left ventricular internal diameter at end-diastole (RVID,d/LVID,d), and right ventricular wall thickness (RVWT), as well as a significant decrease in tricuspid annular plane systolic excursion (TAPSE) in rats exposed to hypoxia for 14d and 28d (Fig. 2A,C,D). Both cardiac output (CO) and cardiac index (CI) of right ventricle were significantly increased after 14d and significantly decreased CO after 28d of hypoxia (Fig. 2A,E). Coronary flow reserve (CFR) significantly decreased in rats exposed to hypoxia for 14d and 28d (Fig. 2F).

The left ventricular isovolumic relaxation time (IVRT) was significantly extended in rats following 28d of hypoxia, isovolumic contraction time (IVCT) was significant decreased and aortic ejection time (AET) was significant increased after 14d of hypoxia, with unchanged after 28d of hypoxia (Fig. 2A,G). The significantly decreased early diastolic mitral annular velocity (e'), ratio of mitral valve early diastolic velocity (MV E) to mitral valve atrial contraction velocity (MV A), the ratio of e' to late diastolic mitral annular velocity (e'/a') and the significantly increased E/e' were observed in rats exposed to hypoxia for 14d and 28d (Fig. 2A,H). Left ventricular (LV) M-mode echocardiographic images in short-axis view suggested significantly decreased left ventricular anterior wall thickness at end-diastole (LVAW,d) and at end-systole (LVAW,s) after 14d of hypoxia (Fig. 2A,I). Parasternal long-axis (pLAX) B-mode imaging revealed that left ventricular volume at end-diastole significantly decreased after chronic hypoxia (Fig. 2A,I). LV mass significant decreased in 14d of hypoxia (Fig. 2A,J). However, no significant difference was observed after 28d of hypoxia, suggesting myocardial cell loss at 14d of hypoxia. Ejection fraction (EF) and fractional shortening (FS), indicators of LV global systolic function, significantly decreased in rats exposed to hypoxia for 14d and 28d (Fig. 2A,K). In addition, we found a significant decrease in LV global longitudinal strain (GLS) (Fig. 2A,L), and radial strain (RS), radial strain rate (RSR), longitudinal strain (LS), and longitudinal strain rate (LSR) of the long axis in rats exposed to hypoxia for 14d and 28d (Fig. 2M).

### Exposure to hypoxia resulted in myocardial cell injury, hypertrophy, fibrosis, lipid deposition, apoptosis, and mitochondrial dysfunction in rats

**Figure 3:**
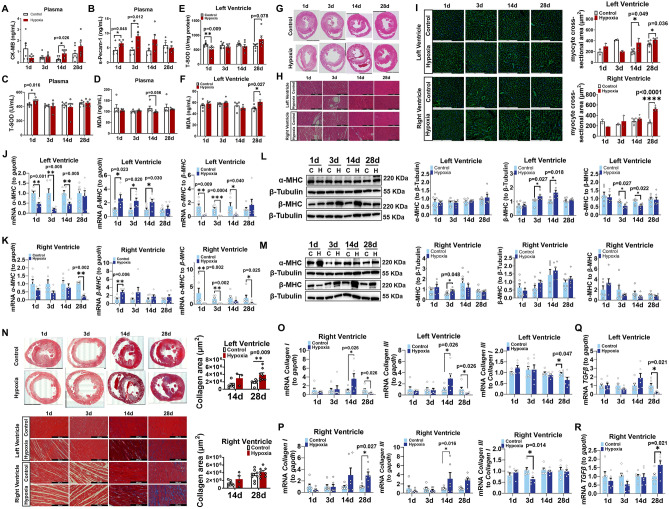
Hypoxic resulted in myocardial injury, hypertrophy, and fibrosis in rat ventricles. (**A**–**D**) Plasma CK-MB, s-Pecam-1, T-SOD, and MDA levels. (**E**,**F**) T-SOD and MDA levels in left ventricular myocardial tissues. (**G**,**H**) HE staining of global cardiac sections (scale bar = 5 mm) and local myocardial cells (scale bar = 200 μm). (**I**) WGA staining (scale bar = 200 μm). (**J**,**K**) Quantification of *αMHC*, *βMHC* mRNA levels and *αMHC*/*βMHC* ratio in left and right ventricular myocardial tissues. (**L**,**M**) Western blot analysis of αMHC, βMHC, αMHC/βMHC ratio in left and right ventricular myocardial tissues. (**N**) Masson staining (scale bar = 200 μm). (**O**,**P**) Quantification of *Collagen I* and *Collagen III* mRNA levels and the *Collagen III/Collagen I* ratio in left and right ventricular myocardial tissues. (**Q**,**R**) Quantification of *TGFβ* mRNA levels in left and right ventricular myocardial tissues. The reference gene was *Gapdh*. n = 6. Results are presented as mean ± SEM. The *p* values are shown on the top of compared group by two-tailed unpaired t-test (for normally distributed data) or Mann–Whitney U test (for nonnormally distributed data).

The plasma CK-MB levels were significantly increased in rats exposed to hypoxia for 14d (Fig. 3A, n = 6), while plasma s-Pecam-1, the marker of endothelial cell proliferation and angiogenesis, were significantly increased in rats exposed to hypoxia for 1d and 3d (Fig. 3B), indicating that systemic endothelial cell damage occurred earlier than myocardial cell damage under hypoxia. The plasma antioxidant total superoxide dismutase (T-SOD) concentration was significantly increased after 1d of hypoxia (Fig. 3C), while malondialdehyde (MDA) concentration was not significantly changed (Fig. 3D). LV T-SOD levels were reduced after 1d of hypoxia (Fig. 3E), and LV MDA levels were elevated after 28d of hypoxia (Fig. 3F). Hematoxylin and eosin (HE) staining revealed right ventricular enlargement and thickened right ventricular walls in rats chronically exposed to hypoxia, consistent with the findings of echocardiology (Fig. 3G). Severe disarrangement of myocardial cells, myocardial cell hypertrophy, and increased vacuolated cells were noted in rats after 14d and 28d of hypoxia (Fig. 3H).

Wheat germ agglutinin (WGA) staining demonstrated significantly increased average cross-sectional area of LV myocardial cells after 14d and 28d of hypoxia and of RV myocardial cells in rats after 28d of hypoxia (Fig. 3I). The myosin heavy chain (MHC) serves as a marker of myocardial cell hypertrophy^27^. After 1d, 3d, and 14d of hypoxia, LV *α-MHC* mRNA significantly decreased, *β-MHC* mRNA increased, and the ratio of *αMHC* to *βMHC* was significantly decreased (Fig. 3J). The changes in RV *α-MHC* and *β-MHC* mRNA levels were similar to changes observed in LV myocardial tissues, with significant changes occurred in 28d of hypoxia (Fig. 3K). Increased protein expression levels of β-MHC and decreased ratio of α-MHC/β-MHC in LV were observed in hypoxic rats after 3d and 14d of hypoxia (Fig. 3L), and the protein expression levels of α-MHC in RV were significant increased after 3d of hypoxia (Fig. 3M). Masson's trichrome staining suggested collagen components and fibrosis were significant increased after chronic hypoxic exposure for 14d and 28d (Fig. 3N). *Collagen I* and *Collagen III* mRNA levels were significantly increased in LV after 14d of hypoxia (Fig. 3O) and significantly increased in RV after 28d of hypoxia (Fig. 3P). However, the protein levels of Collagen I and Collagen III were not altered (Fig. S3A–D). The mRNA levels of Transforming growth factor β (*TGFβ*), a stimulator of myocardial fibrosis, significantly decreased in LV and increased in RV after 28d of hypoxia (Fig. 3Q,R).

**Figure 4:**
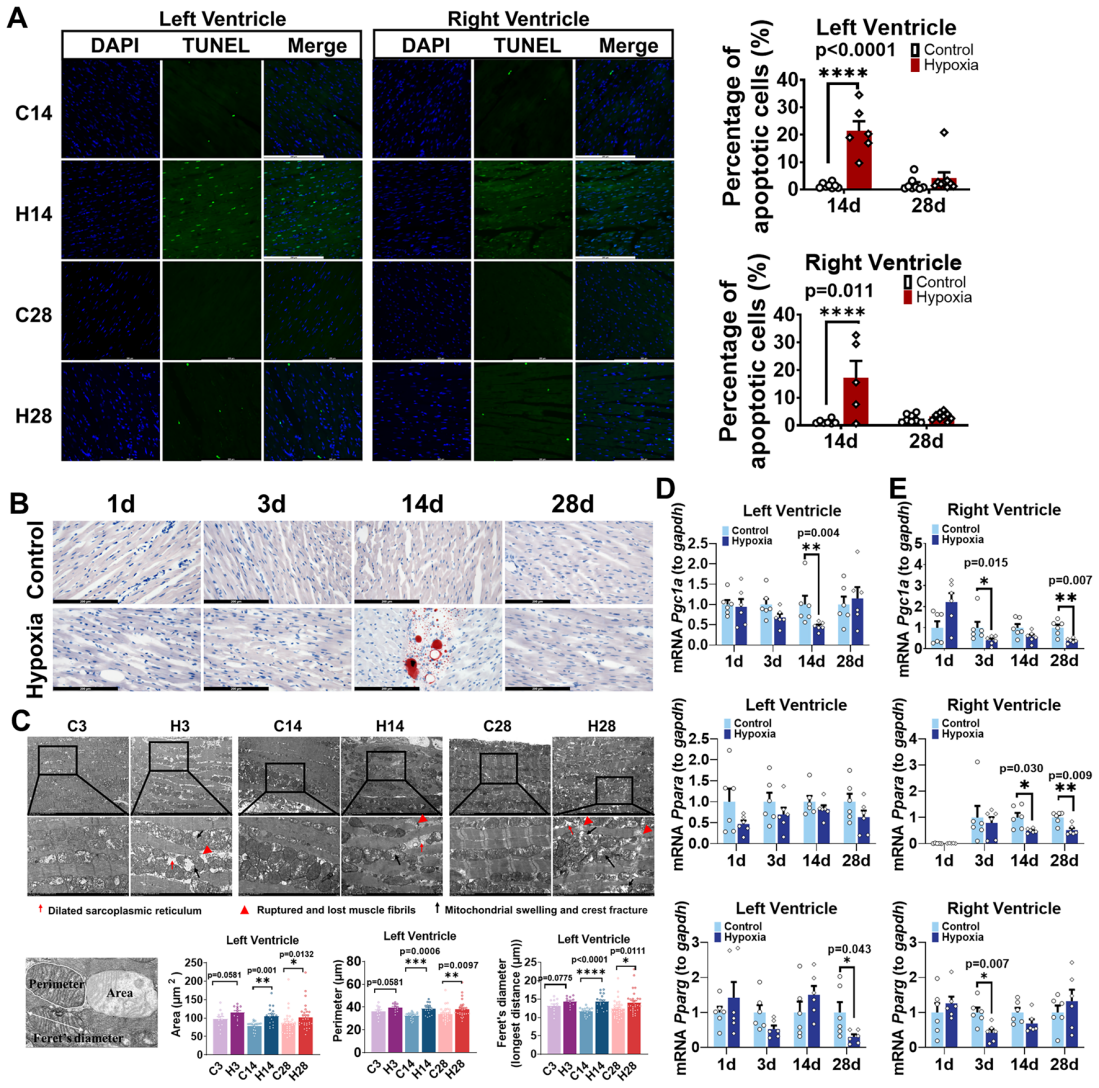
Hypoxic increased apoptosis, lipid deposition, and decreased mitochondrial metabolic in rat myocardial tissues. (**A**) TUNEL staining of left and right ventricles (scale bar = 200 μm). (**B**) Oil Red O staining of left ventricular myocardial tissues (red: lipids, scale bar = 200 μm). (**C**) Upper: Transmission electron microscopy image of left ventricular myocardial tissues. Bottom: Quantification of mitochondrial perimeter, area, and maximum diameter. (**D**,**E**) Quantification of *Pgc1a*, *Ppara*, and *Pparg* mRNA levels in left and right ventricular myocardial tissues. n = 6. Data are presented as mean ± SEM. The *p* values are shown on the top of compared group by two-tailed unpaired t-test (for normally distributed data) or Mann–Whitney U test (for nonnormally distributed data).

Terminal deoxynucleotidyl transferase dUTP nick end labeling (TUNEL) staining revealed significantly increased apoptosis in the left and right ventricular myocardium after 14d of hypoxia (Fig. 4A), but the difference was not significant after 28d of hypoxia. These findings suggest that myocardial cell apoptosis occurs mainly after 14d of hypoxia, which is consistent with the decreases in LV mass and wall thickness. Oil Red O staining revealed significant fibrofatty infiltration in LV after 14d of hypoxia (Fig. 4B). Transmission electron microscopy revealed abnormal mitochondria with swelling, incomplete membranes, disordered arrangements of cristae, and increased electron density in mitochondrial matrix of LV myocardial cells of rats exposed to hypoxia for 3d. Additionally, disordered arrangement and local dissolution of myofilaments, dissolution of Z bands and H bands, and dilated sarcoplasmic reticulum tubules were observed at 14d and 28d in hypoxic rats (Fig. 4C). The area, perimeter, and Feret diameter of mitochondria from 5 randomly selected regions were analyzed and were found to be significantly increased in the 3d, 14d, and 28d hypoxic rats (Fig. 4C). In LV myocardial tissues, *Pgc1α* mRNA was significantly decreased in 14d hypoxic rats and *Pparg* mRNA was significantly decreased in 28d hypoxic rats (Fig. 4D). In RV myocardial tissues, *Pgc1α* and *Pparg* mRNA were significantly decreased in 3d hypoxic rats, *Pgc1α* and *Ppara* mRNA were significantly decreased in 28d hypoxic rats, and *Pparg* mRNA was significantly decreased in 3d hypoxic rats (Fig. 4E).

### Hypoxic exposure led to a reduction in Leptin/Ob-Rb and downstream JAK2/STAT3, PI3K/Akt, and MAPK pathway expression in rat biventricular myocardial tissues

**Figure 5:**
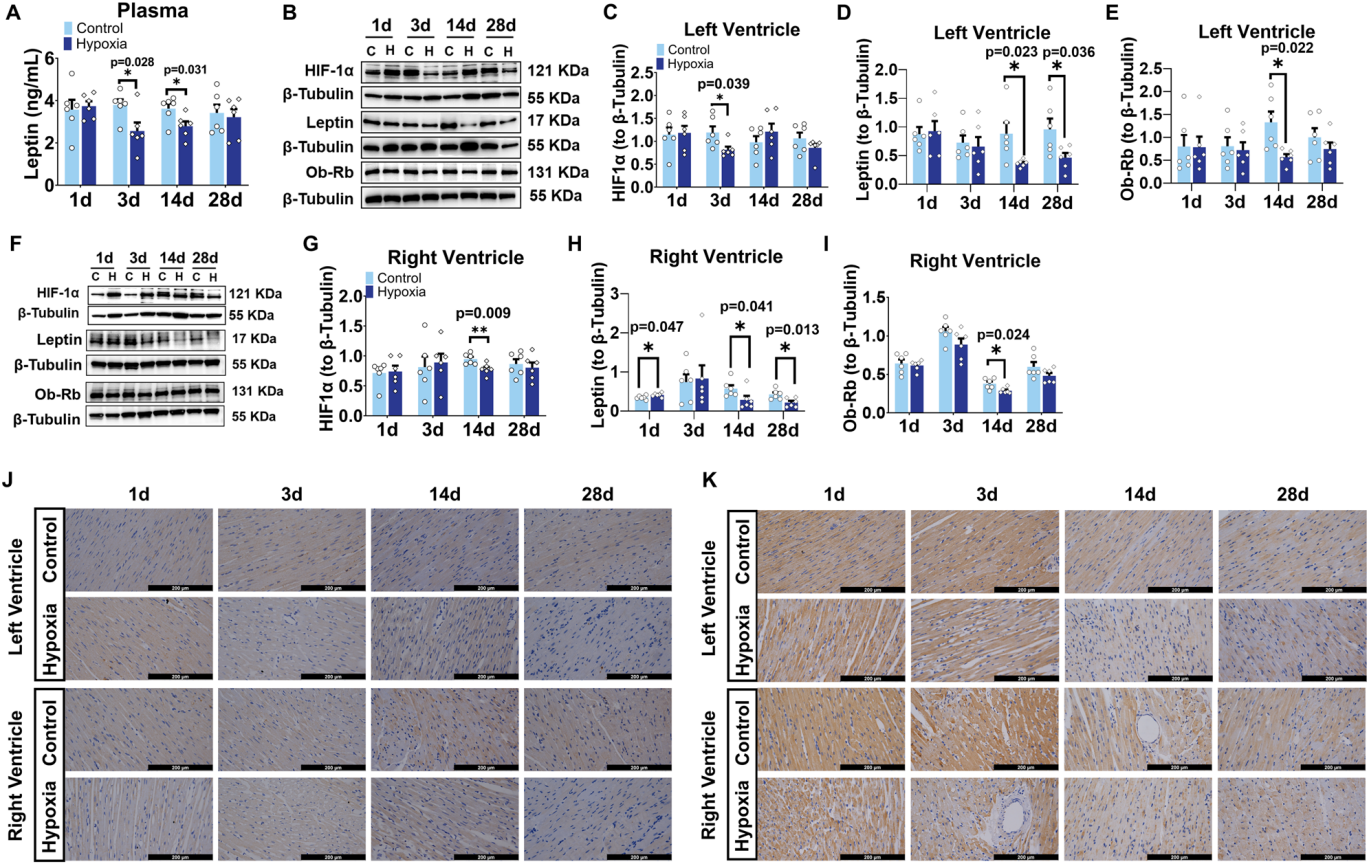
Hypoxia resulted in decreased leptin, ob-Rb, and HIF1α protein levels in rat myocardial tissues. (**A**) Plasma leptin. (**B**) Western blot analysis of HIF1α, leptin, and Ob-Rb in left ventricular myocardial tissues. (**C**–**E**) Quantification of HIF1α, leptin, and Ob-Rb protein expression in left ventricular myocardial tissues. (**F**) Western blot analysis of HIF1α, leptin, and Ob-Rb in right ventricular myocardial tissues. (**G**–**I**) Quantification of HIF1α, leptin, and Ob-Rb protein expression in left ventricular myocardial tissues. The protein expression levels were normalized to those of β-Tubulin. (**J**,**K**) Immunohistochemistry of leptin and Ob-Rb in left and right ventricular myocardial tissues. n = 6. Data are presented as mean ± SEM. The *p* values are shown on the top of compared group by two-tailed unpaired t-test (for normally distributed data) or Mann–Whitney U test (for nonnormally distributed data).

To further investigate the mechanisms underlying hypoxia-induced myocardial injury, we quantified leptin levels in plasma and heart tissues. Compared to those of control group, plasma leptin levels in hypoxia group were significantly decreased after 3d and 14d, and no longer reduced after 28d (Fig. 5A, n = 6). The protein expression of hypoxia inducible factor-1 (HIF1α) was significantly decreased in LV after 3d of hypoxia and in RV after 14d of hypoxia (Fig. 5B,C,F,G, n = 6). The protein expression of leptin significantly increased in RV after 1d of hypoxia and significantly decreased in left and right ventricles after 14d and 28d of hypoxia (Fig. 5B,D,F,H). The long form of leptin receptor (ob-Rb) and in the left and right ventricles significantly decreased after 14d of hypoxia (Fig. 5B,E,F,I). Immunohistochemical results also showed that leptin and ob-Rb levels in LV and RV myocardial cells decreased after 14d of hypoxia (Fig. 5J,K).

**Figure 6:**
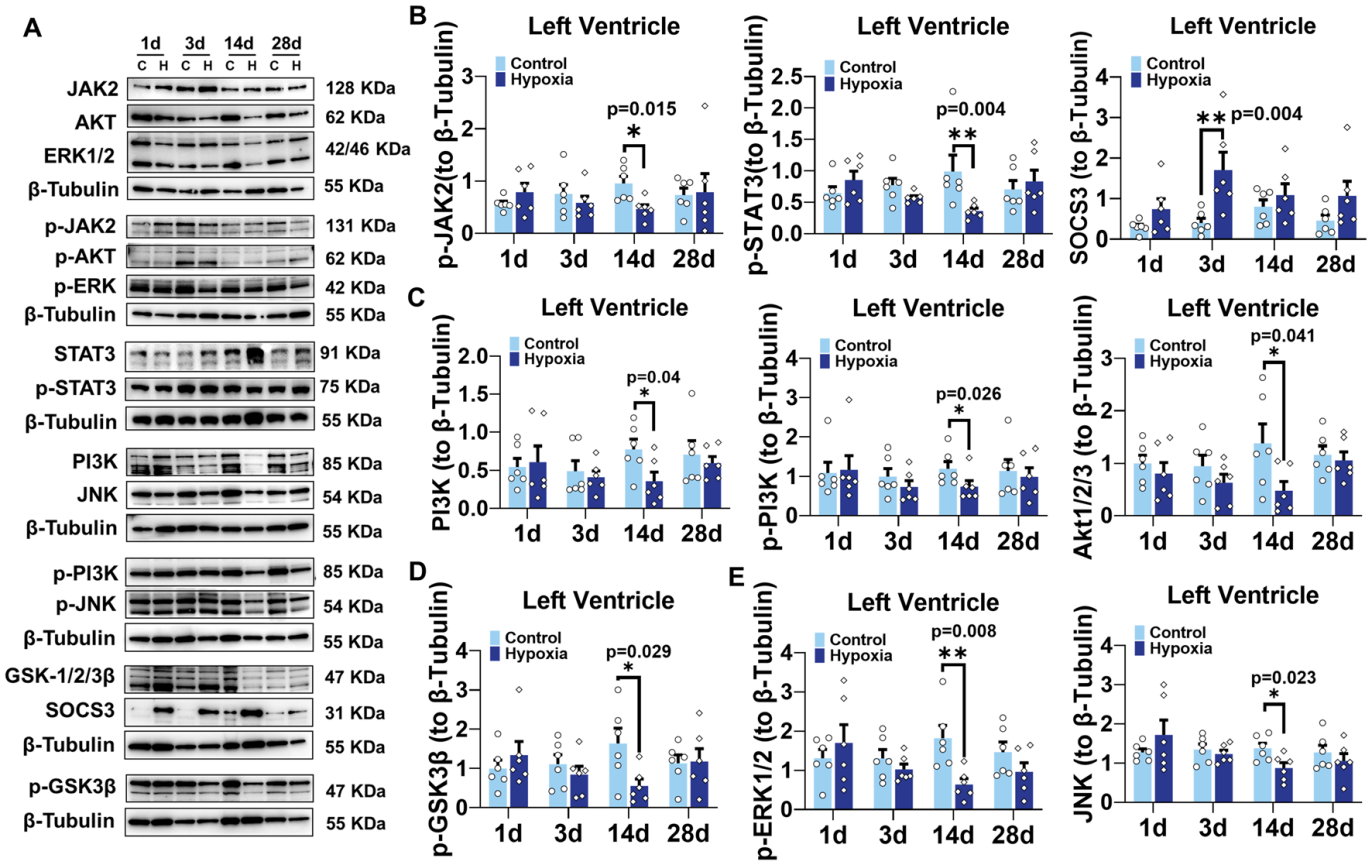
Hypoxia attenuates the expression of leptin signaling pathway proteins in rats myocardial tissues. (**A**) Western blot analysis of JAK2, p-JAK2, STAT3, p-STAT3, SOCS3, ERK1/2, p-ERK1/2, JNK, p-JNK, PI3K, p-PI3K, AKT, p-AKT, GSK3β, and p-GSK3β in left ventricular myocardial tissues. (**B**) Protein levels of p-JAK2, p-STAT3, and SOCS3 in left ventricular myocardial tissues. (**C**) Protein levels of PI3K, p-PI3K, Akt, in left ventricular myocardial tissues. (**D**) Protein levels of p-GSK3β in left ventricular myocardial tissues. (**E**) Protein levels of p-ERK1/2 and JNK. The protein expression normalized to β-Tubulin. n = 6. Data are presented as mean ± SEM. The *p* values are shown on the top of compared group by two-tailed unpaired t-test (for normally distributed data) or Mann–Whitney U test (for nonnormally distributed data).

To elucidate the potential mechanism of leptin in hypoxia-induced myocardial injury, we investigated the dynamic alterations in leptin-induced classical pathways in myocardial tissues. The protein levels of p-JAK2, p-STAT3, total PI3K, p-PI3K, Akt1/2/3, p-GSK3β, p-ERK1/2, and total JNK in LV were significantly decreased after 14d of hypoxia (Fig. 6A–E, n = 6). Whereas, there were no significant differences in total JAK2, p-JAK2 to total JAK2, total STAT3, p-STAT3 to total STAT3, p-PI3K to total PI3K, p-Akt1/2/3, p-Akt1/2/3 to total p-Akt1/2/3, total GSK3β, p-GSK3β to total GSK3β, total ERK1/2, p-ERK1/2 to total ERK1/2, p-JNK, and p-JNK to total JNK protein levels (Fig. 6A, Fig. S3E). The expression of suppressor of cytokine signaling 3 (SOCS3), a key downstream molecule and a critical inhibitory factor of the JAK2/STAT3 signaling pathway, was found to increase significantly after 3d of hypoxia (Fig. 6A,B).

Overall, hypoxic exposure significantly increased myocyte hypertrophy, fibrosis, fat infiltration, apoptosis and mitochondrial dysfunction in myocardial tissues. These changes might affect ventricular contractile and diastolic functions, with the most severe pathological changes occurring at 14 days. Importantly, the protein expression of leptin/ob-Rb signaling pathway molecules in myocardial tissues significantly decreased after 14 days of hypoxic exposure. Noticeable, LV mass, IVCT, AET, plasma CK-MB, α-MHC and β-MHC mRNA, cell apoptosis were all unchanged after 28d, and the LV T-SOD and LV MDA increased after 28d, which revealed the left ventricular may develop a compensatory mechanism to adapt to hypoxia and make up for make up for its inadequate pumping function.

### Leptin was associated with genes involved in circadian rhythm, sodium–potassium ion channel transport, and cytoskeleton

**Figure 7:**
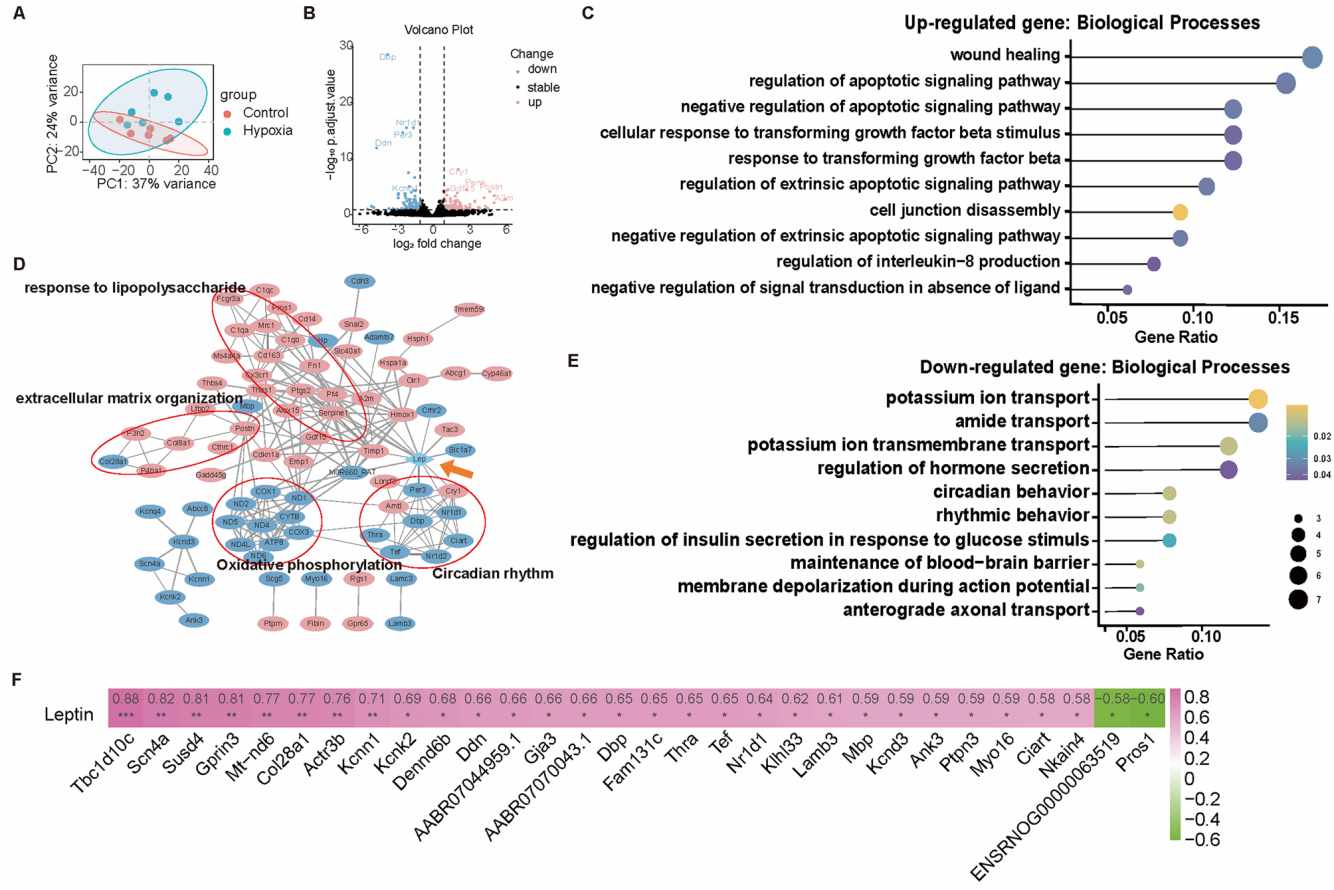
Transcriptomic analysis of left ventricular myocardial tissues in rats and the association with leptin. (**A**) Principal component analysis of the left ventricular myocardial transcriptomes of the 14-day groups. (**B**) Volcano plot of differentially expression genes (DEGs) according to at least twofold change (adjusted *p* value less than 0.05). (**C**,**D**) Enrichment analysis of upregulated and downregulated genes. The top 10 Gene Ontology terms were shown ranked by gene ratio. The circle size represents numbers of gene involved. (**E**) Protein–protein interaction analysis network diagram of DEGs and leptin. Genes in pink circle are upregulated genes and genes in blue circle are downregulated genes. Leptin is rhomboid. (**F**) Pearson correlation analysis between DEGs and leptin. The numbers indicate the correlation coefficient. *p < 0.05. n = 6.

To investigate the potential mechanism through which leptin affects cardiac function after 14d of high altitudes, we performed RNA-seq analysis on LV myocardial tissues from 14d groups. Compared to that in the normoxic group, the gene expression pattern in LV was altered after 14 days of hypoxia (Fig. 7A, n = 6), revealing 158 significantly differentially expressed genes (DEGs; adjusted *p* value less than 0.05 with a fold change more than 2), with 72 upregulated genes and 86 downregulated genes (Fig. 7B, Fig. S4A). Gene enrichment analysis revealed that the upregulated genes were enriched in regulation of apoptotic signaling pathway, cellular stress and inflammatory response to TGF-β and IL8, cell junction disassembly, fibroblast proliferation regulation, response to glucocorticoids, and so on (Fig. 7C). The downregulated genes were enriched in circadian rhythm behavior, potassium ion transport, regulation of insulin secretion, and membrane depolarization during action potential (Fig. 7D).

Protein–protein interaction (PPI) analysis (Fig. 7E) suggested that leptin was associated with proteins encoded by circadian rhythm-related genes (*Cry1*, *Nr1d1*, *Per3*, *Arntl/Bmal1*), genes involved in inflammation and oxidative stress, angiogenesis, cell proliferation and apoptosis (*Crhr2*, *Serpine1*, *A2m*, *Hmox1*, *Ptgs2*, *Tac3*, *Timp1*, *Pf4*), glutamate transport-related genes (*Slc1a7*), and glucose metabolism and glycolysis-related gene (*M0R660RAT*). Furthermore, Pearson correlation analysis (Fig. 7E, Fig. S4B) revealed that leptin was significantly positively correlated with genes involved in circadian rhythm (*Ciart*, *Dbp*, *Nr1d1*, *Tef*), sodium/potassium transport dependent on calcium/calmodulin-dependent kinase II (CaMKII) (*Tbc1d10c*, *Nkain4*, *Kcnd3*, *Kcnn2*, *Kcnn1*, *Scn4a*, *Thra*), cytoskeleton and cell cycle (*Ptpn3*, *Myo16*, *Gja3*, *Actr3b*, *Ank3*), extracellular matrix protein (*Lamb3*, *Col28a1*), mitochondrial respiratory chain and oxidative phosphorylation (*Mt-nd6*, *M0R660RAT*), neurites (*Gprin3*, *Mbp*), and complement activation inhibitor (*Susd4*).

## Discussion

We reported significant reduced plasma leptin levels in expeditioners after 20 days sojourn at Antarctica's Kunlun Station (4087 m), and a similar decline was observed in plasma and myocardial tissue leptin levels in rats exposed to hypobaric hypoxia (5000 m). Leptin is almost exclusively produced by adipose tissues (95%)^28^ and the targeted gene of HIF1α^29^. Exposure to high altitudes significantly affects serum leptin levels, but the results are inconsistent. It seems that acute high altitudes (less than 7 days) exposure led to blood leptin unchanged or increased^20–22^. While prolonged high altitudes exposure led to blood leptin decreased^23,24^. The decrease in blood leptin under prolonged hypoxia may be the result of loss of weight and fat mass or reduction of fat secretion^25^.

After staying on the Antarctic Plateau for 20 days, expeditioners displayed markedly elevated heart rate, blood pressure, and systemic vascular resistance, which may be caused by sympathoexcitation and an increase in pulmonary artery pressure^5^. Studies noted a significant rise in systemic vascular resistance and leg vascular resistance in participants after acclimatization at 4300 m for 21 days^30^. Changes in biventricular mechanics and vascular resistance paralleled the increase in plasma catecholamine concentrations^30,31^. The decreased LV contractile and pumping performance may be due to a reduction in blood volume^5^, but another study revealed that the reduced plasma volume was offset by the increase in erythrocyte volume^30^. LVET, PEP, and STR are three clinical indicators that serve as effective screening tools for left ventricular diastolic dysfunction more than systolic dysfunction^32^. The increased STR may be due to the decreased velocity of myocardial fiber shortening during the ejection period^33^. Notably, the QTc interval was also significantly prolonged by half of expeditioners (over 440 ms), indicating increased cardiovascular morbidity risk^34^.

Comprehensive transthoracic echocardiography of hypoxic rats also revealed chronic hypoxia induced PAH, RVH, loss of LV mass, and left ventricular systolic and diastolic mildly reduced. The decrease in coronary flow reserve further substantiates the progression of hypoxia-induced ventricular dysfunction. Acute high-altitude climbing (4559 m) also resulted a 2–threefold increase in PAP, leading to left ventricular diastolic dysfunction with decreased E/A and e'/a', which were directly related to the severity of PAH^35^. Our results also suggested that chronic hypoxia significantly decreased GLS with decreased regional longitudinal strain, which is a measure of the ability of myocardium to elongate, shorten, thicken, and rotate^36^. Decreased LV GLS has also been observed during high-altitude climbing (4808 m) conditions under gradual hypoxic exposure^37^. The decrease in α-MHC and increase in β-MHC also suggested the disruption of myocardial fibers, which may also contribute to cardiac dysfunction. Compared with fast-twitch fibers (αMHCs), slow-twitch fibers (βMHCs) have a slower lengthening contraction force and lower ATP consumption^38^. Phosphocreatine/adenosine triphosphate (PCr/ATP) was significantly decreased in healthy individuals at the base camp of Mount Everest (5300 m), which is associated with decreased LV mass and diastolic function, suggesting that insufficient energy supply to the heart may be a potential cause of hypoxia-induced cardiac dysfunction^4^.

Our study demonstrated that chronic hypoxia induced cardiomyocyte hypertrophy, apoptosis, oxidative stress, and myocardial fibrosis, with these pathological changes peaking after 14 days. Noticeably, the changes of CK-MB, α-MHC and β-MHC mRNA, and cell apoptosis were gradually recovered after 28 days of hypoxic exposure, coinciding with the decline of leptin and downstream signaling pathways. Accumulation of collagen fibers in the extracellular matrix (ECM) revealed myocardial tissues fibrosis and cardiac remodeling, potentially causing left ventricular stiffness and lower compliance^39^. Lipid deposition, mitochondrial dysfunction, and downregulated expression of *Pgc1α*, *Ppara*, and *Pparg* genes indicated an imbalance in cardiac energy metabolism and mitochondrial homeostasis. In vivo^40^ and in vitro^41–43^ studies have explored leptin's protective role against myocardial ischemia, where exogenous leptin supplementation activates PI3K/Akt, MAPK pathways, and JAK2/STAT3 signaling pathway, reducing mitochondrial permeability transition pore (MPTP) opening, restoring cardiac glucose metabolism, improving cardiac electrophysiological activity, and lowering the expression of pro-inflammatory genes^40–45^. Myocardial tissue-specific knockout mice (*ob*^*−/−*^) exhibited a diminished activity of STAT3 and PI3K/Akt signaling pathways, correlating with cardiac dysfunction, left ventricular dilation, and heightened oxidative stress^45^.

Hyperleptinemia in obese or diabetic patients and increased leptin in epicardial adipose tissue (EAT) have been associated with impaired cardiac function, endothelial oxidative stress, fibrosis, and vascular inflammation^46,47^. In contrast, *ob/ob* mice and *db/db* mice exhibit left ventricular hypertrophy (LVH), increased myocardial lipotoxicity^48^, and cardiac systolic and diastolic dysfunction^44,49^. Leptin supplementation significantly improved cardiac function and reduced lipid deposition in *ob/ob* mice^49^. Moreover, leptin treatment inhibited apoptosis^50^, inflammation, and oxidative stress^41^, and improved cardiac electrophysiology in myocardial ischemic mice and ischemic cardiomyocytes^40^. In contrast to the obesity observed in *ob/ob* and *db/db* mice, hypoxia results in weight and fat loss, resembling the extreme conditions observed in lipodystrophy diseases, which induce a high risk of cardiovascular diseases and hypoleptinemia^51^. Lipodystrophy and obesity represent two contrasting conditions to underscore the connection between leptin and cardiovascular health. Although no current model adequately reconciles the contradictory findings on the effects of leptin on cardiovascular function (Table S1), our study provided a new insight from hypoleptinmia caused by hypoxia and suggested that maintaining circulating leptin within a certain physiological range may be necessary to avoid metabolic imbalances and cardiovascular dysfunction.

The association between leptin and clock genes suggested that *Nr1d1*, *Ciart*, *Dbp*, and *Tef* may serve as the molecular link between leptin and the regulation of cardiac rhythm genes, thereby influencing cardiovascular health^52^. The heart is a peripheral circadian oscillator regulated by core clock genes, including *Clock*, *Bmal1*, and *Nr1d1*^53^. *Nr1d1* is directly suppressed by *Bmal1*, *Clock*, and *Cry1* and participates in metabolism, inflammation, and cardiovascular diseases^54^. *Nr1d1* regulates cardiac inflammasome in cardiac fibroblasts and protects heart against myocardial ischemia–reperfusion injury^55^. *Ciart* functions as a transcriptional repressor of clock genes, inhibiting glucocorticoid response^56^, and is downregulated in cardiac tissues of rats with stress myocardial injury^57^. *Tef* and *Dbp* are homologous and regulated by circadian rhythm. *Dbp/Tef/Hlf* (hepatic leukemia factor) triple gene knockout mice exhibited cardiac hypertrophy and LV dysfunction associated with hypotension^58^. Studies have shown that the rhythms of clock-controlled genes expression were significantly altered and improved by leptin treatment in *ob/ob* mice^59^. Rhythm genes, in turn, regulate leptin expression and metabolism. Mice with *Nr1d1* or *Bmal1* deficiency exhibited impaired food intake, leptin sensitivity and leptin signaling^60,61^.

Furthermore, a significant positive correlation was found between leptin and the down-regulated sodium/potassium transport-related genes *Tbc1d10c*, *Nkain4*, *Kcnd3*, *Kcnk2*, *Kcnn1*, and *Scn4a*, which were also significantly downregulated in cardiac hypertrophy^62^, heart failure^63^, arrhythmias^64^, and atrial fibrillation^65^. However, enhancing the expression of these channel proteins could reduce the myocardial infarct area and cardiac fibrosis^66^ and reverse myocardial hypertrophy^63^. Long-term leptin treatment upregulated the mRNA expression of the subunits of Kv4.2 and Kv4.3 channels (*Kcnd3*) in ventricular myocytes of rats, leading to increased amplitudes and densities of the fast transient outward potassium current, which is mediated by Akt signaling^67^.

Additionally, the downregulated genes *Thra* and *Ank3* were also correlated with leptin. *Thra*, a thyroid (T_3_) hormone receptor alpha receptor (TRα), improved cardiac function in animals with cardiac hypertrophy or heart failure^68^. TRα was found to mediate T_3_-stimulated increase in Kcnd2/3 transcription in rat cardiac myocytes^69^. Mice with a dominant negative mutation in TRα1 showed significantly slowing voltage-activated Ca^2+^ transients and contractions in cardiac myocytes^70^. Ankyrin 3 (Ank3), a key nodal protein, was necessary for the integration of cardiac myofilament and intercalated discs and necessary for compensatory cardiac physiological remodeling in heart failure^71^. Ank3 also targets Nav1.5 and its regulatory protein, CaMKII, which coordinates to intercalated discs^72^. Ank3 also significantly inhibited Kv1.1-mediated currents^73^. Cardiomyocytes with reduced Ank3 expression exhibited abnormal targeting of Nav1.5, and reduced Na^+^ channel current density^74^. Therefore, *Thra* and *Ank3* may also be involved in the regulation of cardiac function and sodium/potassium ion channels in cardiac tissues, despite absence of research findings about direct correlation with leptin.

## Conclusions

Chronic hypoxic factors lead to increased cardiomyocyte hypertrophy, myocardial fibrosis, lipid deposition, cell apoptosis, and mitochondrial dysfunction in cardiac tissues, and induce PVH, RVH, left ventricular systolic and diastolic changes. These pathological changes are most severe after 14 days of hypoxia, and although there is cardiac remodeling and compensatory regulation after 28 days of hypoxia, the global impact of hypoxia on cardiac systolic and diastolic function remains unchanged. We also found that the pathological changes in the heart under hypoxia are associated with a weakening of the leptin/ob-Rb signaling pathway, which is closely linked with genes related to circadian rhythm, sodium/potassium ion transport, and cell cytoskeleton.

Taken together, the coincident evidences from Antarctic expeditioners and hypoxic rats provide a novel insight to find the crucial role of leptin in the impact of hypoxia on cardiac function. Attenuated leptin signaling may be involved in hypoxia-induced cardiac injury, offering new clues for the prevention and treatment of cardiac diseases under high-altitude hypoxia.

However, there are some limitations in the study. There is no direct evidence to confirm whether changes in myocardial leptin levels are due to a reduction in cardiac fat tissue content or influenced by other paracrine pathways. And future studies are needed to elucidate and comprehensively understand the interaction of leptin with circadian rhythm genes and sodium/potassium ion transport channels under hypoxia-induce cardiovascular diseases.

## Materials and methods

### Materials

Human Cardiovascular Disease (CVD) Magnetic Bead Panel—Cardiovascular Disease Multiplex Assay, and Human Adipocyte Magnetic Bead Panel—Endocrine Multiplex Assay were purchased from Millipore^®^ Merch Germany.

Rat Creatine phosphokinase-MB (CK-MB, CSB-E14403r) and platelet-endothelial cell adhesion molecule-1 (s-Pecam-1, CSB-E08134r) were purchased from Cusabio Technology. Leptin (SEA084Ra) was purchased from Nanjing Jiancheng Technology. T-SOD (total superoxide dismutase, E-BC-K020-M) ELISA Kit and MDA (malondialdehyde, E-EL-0060) ELISA Kit were purchased from Elabscience Biotechnology (Wuhan, China).

Leptin antibody (GB112924), Leptin receptor antibody (GB112091), and HIF1α antibody (GB111339) from Wuhan Servicebio Technology. STAT3 (F-2) antibody (sc-8019), p-STAT3 (B-7) antibody (sc-8059), JAK2 (C-10) antibody (sc-390539), JNK (D-2) antibody (sc-7345), p-JNK (G-7) antibody (sc-6254), SOCS-3 (6A463) antibody (sc-73045), Akt1/2/3 (5C10) antibody (sc-81434), p-Akt1/2/3 (C-11) antibody (sc-514032) Santa Cruz Biotechnology, Inc. Leptin antibody (bs-0409R), JAK2 (Tyr1007 + Tyr1008) antibody (bs-2485R), ERK1/2 antibody (bs-2637R), p- ERK1/2 (Thr202 + Tyr204) antibody (bs-3016R), PI3K p85 alpha subunit antibody (bs-0128R), p-PI3K p85 (Tyr467 + Tyr199) antibody (bs-3332R), GSK-3β antibody (bs-0028R), p- GSK-3β (Ser9) antibody (bs-2066R) were purchased from Beijing Bioss Biotechnology. Myh6 (22281-1-AP), myh7 (22280-1-AP), collagen I (14695-1-AP), and collagen III (22734-1-AP) antibodies were purchased from Proteintech Group. β-Tubulin (2H4) antibody was purchased from Abmart Shanghai Co. Ltd.

### Antarctic expedition participants

Twenty-three healthy male participants (average age 31.48 ± 6.45 years, height 173.39 ± 6.34 cm, weight 70.53 ± 9.44 kg; refer to Table S2) from the Chinese Antarctic Expedition were recruited for this study. Expeditioners underwent medical examinations and blood sampling, and informed consent was obtained. The study protocol was performed according to the Declaration of Helsinki, and approved by the Institutional Review Board of the Institute of Basic Medical Sciences Chinese Academy of Medical Sciences (CAMS, No. 2018004).

The detailed schedule of Kunlun Station expedition was shown in Supplementary Fig. 5. The team expeditioners who did not participate in the departure examination and blood sampling were directly excluded from following analysis, and the team expeditioners who did not participate in the Kunlun station site examinations and blood sampling due to work tasks were also excluded from following analysis. All expeditioners undergo medical examinations and blood sample collection under the guidance of professional doctors before departure on October 31, 2014. Since there was one experienced doctor on Kunlun Station, all of the non-invasive examinations were conducted on the 18-20th day on Kunlun Station. Blood samples were obtained at departure and on 20th day at Kunlun Station.

Lung function, cardiovascular function, electrocardiogram, body composition, and microvascular oxygen saturation were examined by a digital spirometer (MasterScope^®^, Jaeger, Germany), noninvasive hemodynamic monitor (NiccomoTM Cardioscreen^®^ 2000, Medis, Germany), 12-lead electrocardiograph (MAC800, GE, USA), Omron Karada Scan Body Composition Monitor (HBF-701, Japan), and a pulse oximeter (Philips DB12, China) according to the manufacturer’s instructions by medical professionals at departure (Shanghai, 5 m) and Kunlun Station (Dome A of Antarctica, 4087 m).

Blood samples obtained at departure and on the 20th day at Kunlun Station were subjected to subsequent analysis of the cardiovascular risk factors creatine phosphokinase-MB (CK-MB), platelet-endothelial cell adhesion molecule-1 (sPecam-1), and adipokines including adiponectin, lipocalin-2, resistin, adipsin, and leptin.

### Rats

The animal studies were approved by the Animal Care and Use Committee of Peking Union Medical College, CAMS (ACUC-A02-2022-039). All experiments were carried out in compliance with the ARRIVE guidelines.

Male-specific pathogen-free Sprague–Dawley (SD) rats (eight weeks old) were purchased from WeitongLihua Experimental Animal Technology (Beijing). The rats were housed in controlled environment with 12:12 dark–light cycle, an average temperature of 25.5 ± 0.5 °C, and an average humidity of 53% ± 5%.

The rats were randomly divided into 8 groups and acclimated to the research facility for one week before treatment. After 1 week of preadaptation, the hypoxic group was exposed to a hypobaric oxygen chamber (FLYDWC50-IC) that mimicked an altitude of 5000 m (399 mmHg), while the control group remained in a normoxic environment (52 m, 760 mmHg) within the same room. The chamber was opened for 20 min per day to record body weight and food intake. The hypoxia and control groups were subjected to critical control of hypoxia alone, and an equal number of rats were fed each squirrel cage and provided with ad libitum access to water and food.

After 1 day (1d), 3 days (3d), 14 days (14d), and 28 days (28d) of hypoxia (n = 9/group) and normoxia (n = 9/group) exposure, deep anesthesia was induced using 3% isoflurane, and plasma samples and heart tissues were collected immediately and stored at − 80 °C until analysis.

### Echocardiography

Comprehensive transthoracic echocardiography in rats of 14 days group and 28 days group uses four principal imaging formats to assess cardiac function: brightness mode (B-mode), motion mode (M-mode), PW Doppler, and tissue Doppler imaging. Images were acquired using a digital Vevo 2100 small animal ultrasound system (FUJIFILM VisualSonics Inc., Toronto, Canada) with a 13- to 24- MHz linear-array transducer (MS-250).

Rats were anesthetized with 3% isoflurane and maintained with 1.0–1.5% in oxygen modulated according to heart rate. Hair was removed from the anterior chest using depilatory creams. Anesthetized rats were maintained body temperature (37 °C) on the thermal platform with limbs fixed to the electrodes to enable recording of ECG signals and a pre-warmed ultrasound transmission gel was applied to the chest. A good ECG signal (with the entire signal visible in the physiological data trace) will assist with correctly marking R-Waves for analysis of strain. Standard parasternal long-axis ultrasound examination was performed using a 13 to 24 MHz linear-array transducer (MS-250). Standard short-axis view of the ventricle was acquired at the level of the papillary muscle view. Parasternal short-axis (SAX) M-mode scans at the midventricular level, as indicated by the presence of papillary muscles in rats, for the assessment of the dimensions and functions of LV. For quantification of right ventricular (RV) function, tricuspid annular plane systolic excursion (TAPSE) was recorded with M-mode. B-mode images display two-dimentional (2-D) views of the heart pulmonary artery short- and long-axis cineloops may be traced in diastole and systole to assess cardiac function, including RVOT, RVIDd, RVID,d/LVID, RVWT measurements. Tissue Doppler imaging (TDI) and mitral valve (MV) Doppler was performed to record mitral annular velocity and mitral inflow curves. Tissues Doppler imaging was used to assess hemodynamic of pulmonary artery. Tissue Doppler tracings in apical 4 chamber view showing the measurements to perform for functional assessment of diastolic function of the LV. Veloticy-time integral (VTI) allowed an estimation of CO and CI. At the end of the procedures, all rats recovered from anesthesia without difficulties and returned to their cages.

Speckle-tracking imaging-based regional LV analysis, this sensitive measure enables an early detection of subtle myocardial defects before global dysfunction in hypoxia hearts in rats. Speckle-tracking echocardiography (STE) was performed in the PLAX view at the B-Mode cine loops to provide the reproducible orbits of myocardial movements for longitudinal strain analyses in rats. Tracing the border of endocardium and epicardium and six segments of myocardium in both the long and short axis. Values generated by strain analysis in the longitudinal dimension are negative, indicative of fiber shortening.

Coronary flow velocity was observed on cine traces of the left proximal coronary artery using pulsed-wave Doppler at baseline and under hyperaemic conditions induced by inhalation of 1.5% and 3.0% isoflurane, respectively. Coronary flow reserve (CFR) is evaluated by the ratio of peak blood flow velocity during hyperaemia and peak blood flow velocity at baseline.

All parameters were measured at least three consecutive cardiac cycles by off-line analysis using the VevoLab software (Version 5.6.0, FUJIFILM VisualSonics Inc., Toronto, Canada). A continuous assessment of pathological remodeling was acquired using high spatial and temporal resolution transducers, superior imaging frame rates (300 frames/s), providing superior resolution (~ 30 um) high-quality examination. The methods refer to the recommendations for cardiac chamber quantification by echocardiography^75^. Careful data interpretation by an experienced, blinded operator to obtain meaningful and reproducible cardiac function data.

### Enzyme-linked immunosorbent assay (ELISA)

Creatine phosphokinase-MB (CK-MB), platelet-endothelial cell adhesion molecule-1 (s-Pecam-1), total superoxide dismutase (T-SOD), malondialdehyde (MDA), and leptin levels in plasma, as well as the T-SOD and MDA levels in cardiac tissues were measured using ELISA kits. All experimental procedures were performed according to the manufacturer's instructions.

### Immunohistochemistry

Rat cardiac tissues and adipose tissues were fixed in 4% paraformaldehyde (PFA) overnight at 4 °C, followed by embedding in paraffin or OCT. The sections were cut to a thickness of 5 μm. After dewaxing and dehydration, the sections were fixed in 4% PFA at room temperature for 20 min, permeabilized with 0.2% Triton X-100 for 20 min, blocked with 10% goat serum at room temperature for 1 h, and then incubated with primary antibodies overnight at 4 °C. The primary antibodies were leptin antibody (1:300) and leptin receptor antibody (1:300). After incubation with goat anti-rabbit IgG secondary antibody (1:300). H&E staining, Masson's Trichrome staining, Oil O red staining, wheat germ agglutinin (WGA) staining, and terminal deoxynucleotidyl transferase dUTP nick end labeling (TUNEL) staining were performed according to the manufacturer's instructions.

All sections were imaged using a Leica DMi8 S Inverted Microscope or Leica DM6B Upright Microscopes. The ImageJ software (ImageJ.org) was used to image analysis. Five to eight different fields (200 ×) were analyzed per sample in a double-blind manner by a pathologist.

### Transmission electron microscopy

The left ventricular myocardial tissue was cut into 1 mm^3^ pieces, fixed with 2.5% glutaraldehyde and 1% OsO_4_, dehydrated gradually with ethanol, and embedded in EMBed 812. Ultra-thin sections (80 nm) were double stained with 2% uranyl acetate and 2.6% lead citrate. Mitochondrial ultrastructural changes were observed under a Hitachi HT7700 transmission electron microscope (RuliTEM HT7800).

### Quantitative real-time polymerase chain reaction

Total RNA was purified from frozen myocardial tissue using the Total RNA Extraction Reagent Kit according to the manufacturer's instructions. cDNA synthesis was performed using the EasyScript^®^ First-Strand cDNA Synthesis SuperMix (AE301, TransGen Biotech). Real-time quantitative PCR analysis was conducted using the 2 × Universal Blue SYBR Green qPCR Master Mix (Servicebio Technology), and the primers for target genes are listed in Table S3 (Tsingke Biotechnology). Analysis was performed using the CFX Connect Real-Time PCR Detection System (Bio-Rad Laboratories). GAPDH was used as the reference gene, and the relative mRNA levels of the target genes were determined using the cycle threshold method. The data were reported as the expression levels relative to the reference gene using the 2^−ΔΔCT^ method.

### Western blotting

Total protein of myocardial tissues was extracted using Total Protein Extraction Reagent Kit and then the protein concentration was determined using BCA Protein Assay Kit. Typically, 30–50 µg of total protein from myocardial tissue was loaded onto and separated by sodium dodecyl sulfate (SDS) polyacrylamide gel electrophoresis (4–15%). The proteins were then transferred onto PVDF membranes and blocked with 5% skim milk or 0.1% BSA in tris-buffered saline (TBS) at room temperature for 1 h. The membranes were incubated overnight at 4 °C with primary antibodies. Immunodetection was performed with horseradish peroxidase (HRP)-conjugated anti-mouse or anti-rabbit secondary antibodies at a dilution of 1:3000 at room temperature for 1 h. The HRP-labeled bands were detected using enhanced chemiluminescence (ECL) and imaging system (Tanon 4800 multi), and quantified using Image J. The protein band intensities were normalized to β-tubulin band intensity.

### RNA extraction, sequencing, and analysis

RNA-seq was performed in left ventricular myocardial tissue from hypoxic group of 14d and control group. Total RNA was extracted using the Trizol reagent according to the manufacturer’s protocol (Novogene Co. Ltd., Beijing, China). Sequencing libraries were generated using NEBNext Ultra RNA Library Prep Kit for Illumina (NEB, USA, E7530L) following manufacturer’s recommendations and index codes were added to attribute sequences to each sample. The qualified libraries were pooled and sequenced on Illumina platforms with PE150 strategy in Novogene Bioinformatics Technology Co., Ltd (Beijing, China), according to effective library concentration and data amount required.

Clean paired-end reads with adapter trimming and low-quality read filtering were obtained using fastp (version 0.20.0), and then mapped to the rat genome (Rat rno7.2) using HISAT2 (version 2.1.0). HTSeq (version 0.11.2) was used to calculate gene expression read counts. Differential expression genes (DEGs) were calculated using the *DESeq2* R package (version 1.24.0), with a threshold of FDR (false discovery rate adjusted *p*-value by Benjamini & Hochberg method) < 0.05 and |log_2_fold-change|> 1. Differential gene enrichment analysis was performed using the R package *ClusterProfiler 4.0*, with adjusted *p*-values less than 0.05. Protein–Protein Interaction (PPI) analysis of differential genes and leptin was conducted using *String* (https://string-db.org/) for analysis of the interaction of leptin with proteins coded by DEGs and to predict the potential biological processes involved leptin using *String* enrichment process. RNA-sequencing (RNA-seq) data have been deposited in the Gene Expression Omnibus (GEO; https://www.ncbi.nlm.nih.gov/geo/, GSE245300).

### Statistical analysis

R software (version 4.3.1) was utilized for statistical analysis. Shapiro–Wilk tests were performed to determine the data distribution. For paired data in human studies, the data are presented as the mean ± standard deviation (SD). Comparisons of Kunlun and Departure site data were tested by two-tailed paired t-tests (normal data) or Wilcoxon signed-rank tests (nonnormal data) via the “*rstatix*” package in R. Correlation analysis of paired data was performed by repeated measures correlation (rmcorr) for testing within-individual correlations for paired measures on two occasions for multiple expeditioners using the “*rmcorr*” package in R^76^. The data from independent samples in animal studies are expressed as mean ± standard error of the mean (SEM). Comparisons between two groups were tested by two-tailed unpaired t-tests (normal data) or Mann–Whitney U tests (nonnormal data) via the “*rstatix*” package in R. Pearson correlation analysis of independent data was conducted using the “*Hmisc*” package in R. Graphs were prepared using the “*ggplot2*” package in R and Prism 9.0 software (GraphPad). *p < 0.05 was considered statistical significance.

### Ethics approval and consent to participate

The human study protocol was performed according to the Declaration of Helsinki, and approved by the Institutional Review Board of the Institute of Basic Medical Sciences Chinese Academy of Medical Sciences (No. 2018004). The animal studies were approved by the Animal Care and Use Committee of Peking Union Medical College, Chinese Academy of Medical Sciences (ACUC-A02-2022-039). Written informed consent has been obtained from the participants to publish this paper.

## Supplementary Information

Supplementary Information.

Supplementary Figures.

## Data Availability

The datasets used and/or analysed during the current study are available from the corresponding author on reasonable request.
