# Correction: Association of attenuated leptin signaling pathways with impaired cardiac function under prolonged high-altitude hypoxia

**DOI:** 10.1038/s41598-025-30890-w

**Published:** 2026-01-05

**Authors:** Jianan Wang, Shiying Liu, Lihong Sun, Zhanping Kong, Jiamin Chai, Jigang Wen, Xuan Tian, Nan Chen, Chengli Xu

**Affiliations:** 1 Institute of Basic Medical Sciences, School of Basic Medicine, Chinese Academy of Medical Sciences, Peking Union Medical College, Beijing, 100005 China; 2 Center for Experimental Animal Research, Institute of Basic Medical Sciences, Chinese Academy of Medical Sciences and Peking Union Medical College, Beijing, 100005 China; 3 Qinghai Provincial People’s Hospital, Xining, 810000 Qinghai China; 4 Center of Environmental and Health Sciences, Chinese Academy of Medical Sciences, Beijing, 100005 China

Correction to: *Scientific Reports* 10.1038/s41598-024-59559-6, published online 3 May 2024

The original version of this Article contained an error in Fig. 5K, in which the figure incorrectly uses the immunohistochemistry image of leptin, which should be Ob-Rb. The original Fig. 5 and accompanying legend appear below.

**Fig. 5:**
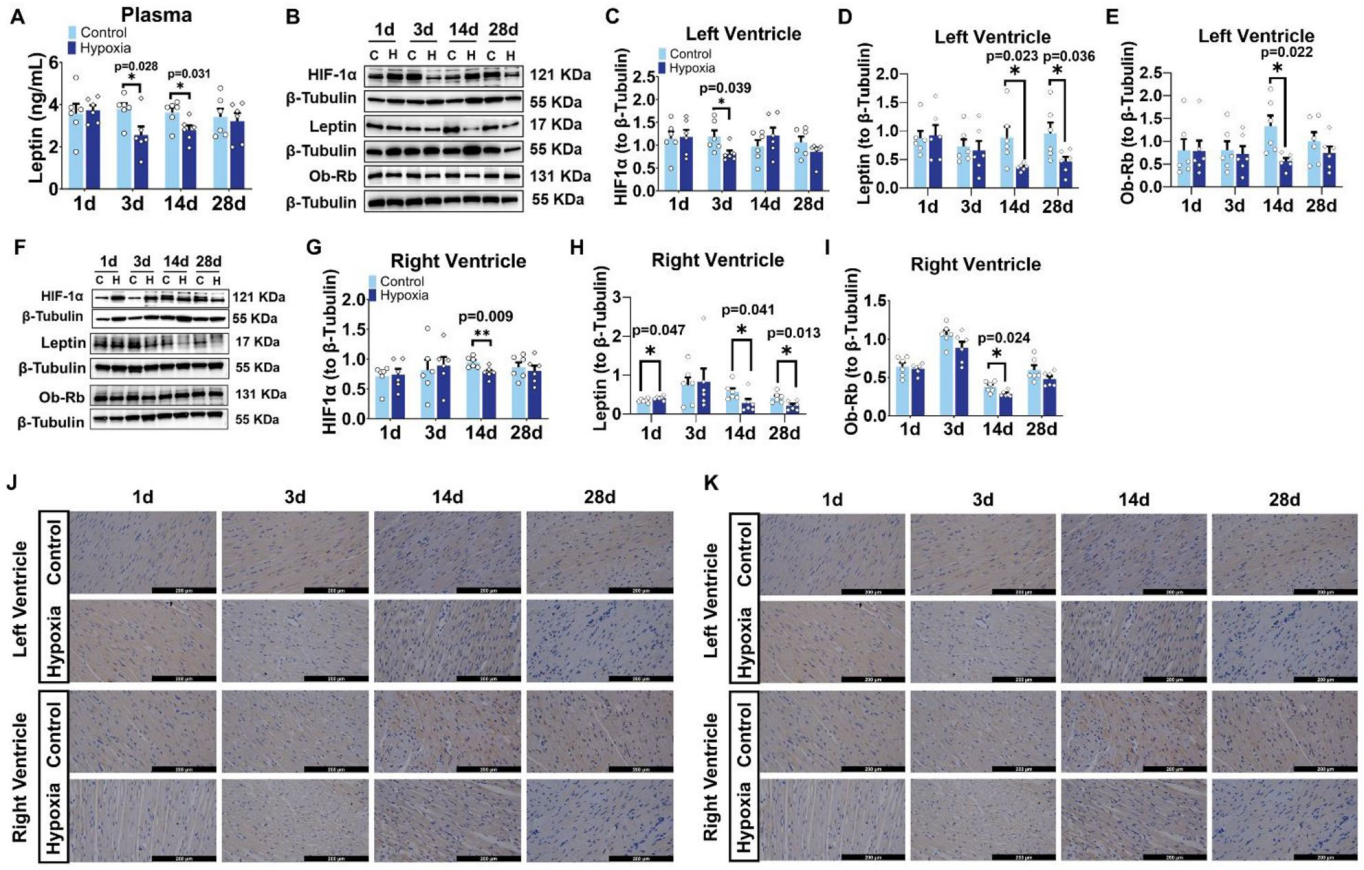
Hypoxia resulted in decreased leptin, ob-Rb, and HIF1α protein levels in rat myocardial tissues. (**A**) Plasma leptin. (**B**) Western blot analysis of HIF1α, leptin, and Ob-Rb in left ventricular myocardial tissues. (**C**–**E**) Quantification of HIF1α, leptin, and Ob-Rb protein expression in left ventricular myocardial tissues. (**F**) Western blot analysis of HIF1α, leptin, and Ob-Rb in right ventricular myocardial tissues. (**G**–**I**) Quantification of HIF1α, leptin, and Ob-Rb protein expression in left ventricular myocardial tissues. The protein expression levels were normalized to those of β-Tubulin. (**J**,**K**) Immunohistochemistry of leptin and Ob-Rb in left and right ventricular myocardial tissues. n = 6. Data are presented as mean ± SEM. The *p* values are shown on the top of compared group by two-tailed unpaired t-test (for normally distributed data) or Mann–Whitney U test (for nonnormally distributed data).

The original Article has been corrected.

