# Supplementary material for: Association of attenuated leptin signaling pathways with impaired cardiac function under prolonged high-altitude hypoxia
Source: Sci Rep. 2024 May 3;14:10206. doi: 10.1038/s41598-024-59559-6 (PMC11068766; doi:10.1038/s41598-024-59559-6)
Supplement: Supplementary file 1 — Supplementary Information. [file 41598_2024_59559_MOESM1_ESM.docx]

**Supplement Table 1. The controversial findings of leptin on cardiac function.**

| Models with treatment | Phenotype | Ref. |
| --- | --- | --- |
| Obese and diabetic male patients | Plasma leptin levels ↑ | ^1^ |
| Cardiomyocyte treated with leptin derived from EAT in obese mice | Apoptosis and oxidative stress ↑ | ^2^ |
| ob/ob mice  db/db mice | Obesity  Cardiomyocyte hypertrophy ↑  Exogenous leptin supplementation reduces body weight, decreases cardiomyocyte hypertrophy | ^3^ |
|  | Cardiomyocyte apoptosis ↑ | ^4^ |
|  | Cardiomyocyte hypertrophy ↑  Left ventricular contractility ↓ | ^5^ |
|  | Myocardial triglyceride ↑ | ^6^ |
| Myocardial infarction mice with leptin supplementation | Myocardial contractile function ↑ | ^7^ |
| Aortic constriction mice with leptin supplementation | Restore action potential  Contractile function ↑  Arrhythmias ↓ | ^8^ |
| Wild-type mice with leptin supplementation | Prolong action potential  Contractile function ↓ | ^8^ |
| Ischemic cardiomyocyte treated with leptin | Apoptosis ↓ | ^9^ |
| Hypoxic cardiomyocytes treated with leptin | Expression of inflammatory genes ↓  Oxidative stress levels ↓ | ^10^ |
| Antarctic expeditioners staying at high altitudes for 20 days | Cardiac contractility ↓  Plasma leptin levels↓ | In this study |
| Hypoxic rats at 5,000 m for 14 days | Cardiac diastolic and contractile function ↓  Plasma leptin levels↓  Cardiac leptin and leptin signaling levels ↓  Cardiomyocyte apoptosis ↑  Hypertrophy and myocardial fibrosis ↑  Mitochondrial metabolic genes ↓ |  |

**Supplement Table 2. Demographic data of participants involved in the study.**

| **ID** | **Age (Years)** | **Gender** | **Height**  **(cm)** | **Weight**  **(kg)** |
| --- | --- | --- | --- | --- |
| **A01** | 23 | Male | 165 | 70.6 |
| **A02** | 24 | Male | 177 | 63.9 |
| **A03** | 26 | Male | 177 | 63.9 |
| **A04** | 24 | Male | 173 | 64.9 |
| **A05** | 39 | Male | 176 | 83 |
| **A06** | 24 | Male | 182 | 63.9 |
| **A07** | 35 | Male | 176 | 79.1 |
| **A08** | 27 | Male | 175 | 67.1 |
| **A09** | 41 | Male | 160 | 67.1 |
| **A10** | 27 | Male | 174 | 65 |
| **A11** | 26 | Male | 184 | 68.5 |
| **A12** | 33 | Male | 169 | 71.3 |
| **A13** | 26 | Male | 168 | 60.1 |
| **A14** | 35 | Male | 182 | 60.6 |
| **A15** | 37 | Male | 173 | 68 |
| **A20** | 33 | Male | 179 | 84.1 |
| **A22** | 33 | Male | 170 | 76.5 |
| **A23** | 30 | Male | 174 | 75.6 |
| **A24** | 39 | Male | 180 | 75.9 |
| **A25** | 27 | Male | 172 | 96.4 |
| **A26** | 45 | Male | 170 | 79.1 |
| **A27** | 30 | Male | 160 | 57.3 |
| **A28** | 40 | Male | 172 | 60.3 |

**Supplement Table 3. The primers template and sequence for target genes.**

| Gene | Primer sequence | | Melt Tempe-rature (℃) | | Template | |
| --- | --- | --- | --- | --- | --- | --- |
| GAPDH | FP | CGGCAAGTTCAACGGCACAGT | 61.15 | NM_017008.4 | |  |
|  | RP | ACGCCAGTAGACTCCACGACAT | 60.68 |  |  |  |
| Col1a1 | FP | ATCAGCCCAAACCCCAAGGAGA | 59.67 | NM_053304.1 | |  |
|  | RP | CGCAGGAAGGTCAGCTGGATAG | 62.38 |  |  |  |
| Col3a1 | FP | TGATGGGATCCAATGAGGGAGA | 57.63 | NM_032085.1 | |  |
|  | RP | GAGTCTCATGGCCTTGCGTGTTT | 61.21 |  |  |  |
| Myh6 | FP | GCCCTTTGACATCCGCACAGAGT | 62.31 | NM_017239.2 | |  |
|  | RP | TCTGCTGCATCACCTGGTCCTCC | 63.28 |  |  |  |
| Myh7 | FP | GCGGACATTGCCGAGTCCCAG | 62.85 | NM_017240.2 | |  |
|  | RP | GCTCCAGGTCTCAGGGCTTCACA | 63.37 |  |  |  |
| TGFβ | FP | GGCTGAACCAAGGAGACGGAAT | 59.53 | NM_021578.2 | |  |
|  | RP | TGGAGCTGTGCAGGTGTTGAG | 60.55 |  |  |  |
| PGC1A | FP | TGGAGTGACATAGAGTGTGCTG | 59.75 | NM_031347.1 | |  |
|  | RP | TATGTTCGCGGGCTCATTGT | 60.11 |  |  |  |
| PPARA | FP | AGACTAGCAACAATCCGCCTT | 59.72 | NM_013196.2 | |  |
|  | RP | GCCTTGGCAAATTCCGTGAG | 60.11 |  |  |  |
| PPARG | FP | ATTATTCTCAGTGGAGACCGCC | 57.95 | NM_001145366.1 | |  |
|  | RP | GCAGCAGGTTGTCTTGGATG | 57.35 |  |  |  |
|  | RP | GGTTCTCGGCTGTGCTGGAATC | 61.15 |  |  |  |

**Supplement Figures**

**
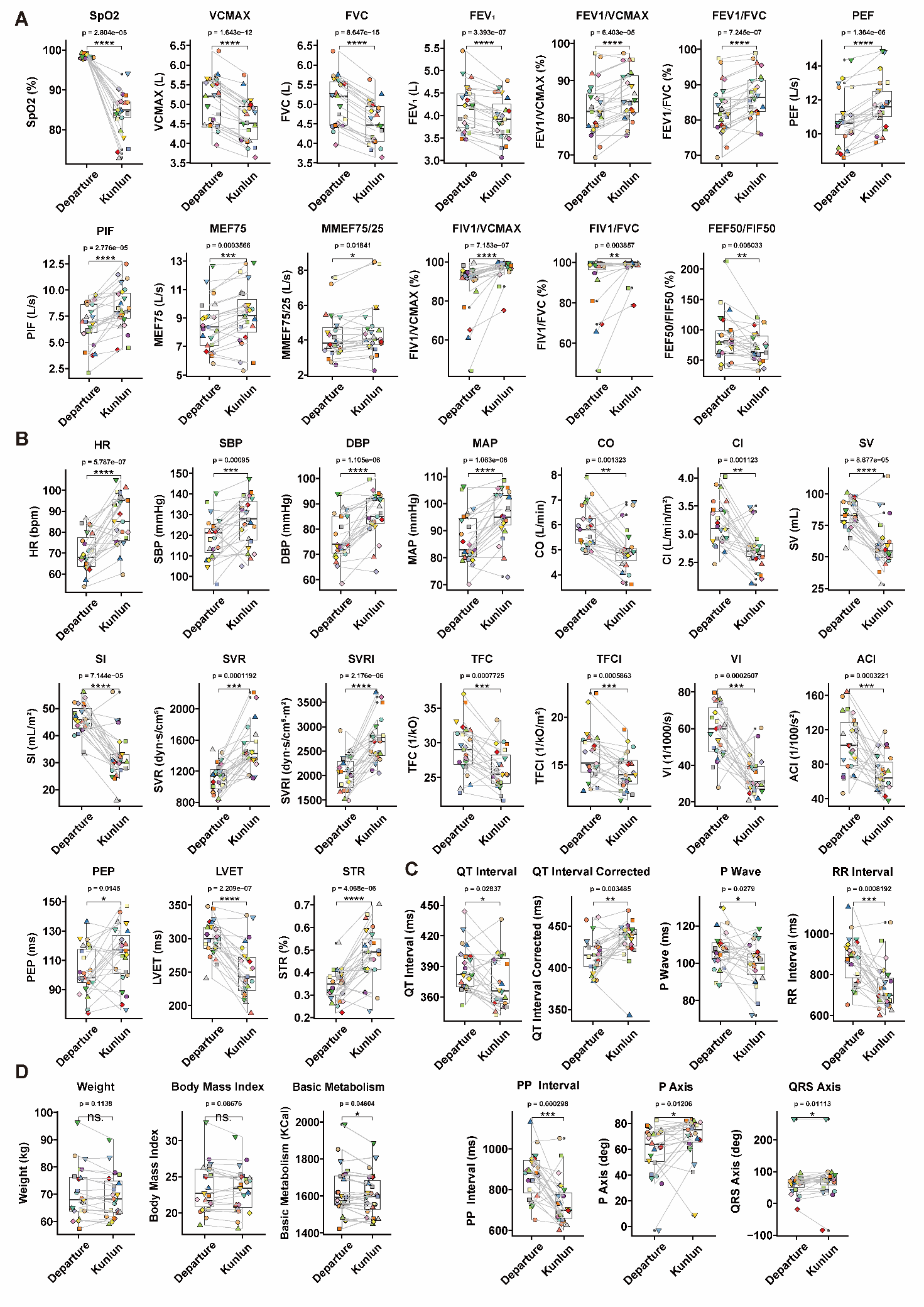
**

**Figure S1. The cardiopulmonary phenotype of the Chinese Antarctic Kunlun Station expedition (N=23). (A). Pulse Oximetry and Pulmonary function. (B). Cardiovascular function. (C). Electrocardiogram. (D). Body composition. P, compared to Departure. VCMAX, vital capacity max; FVC, Forced Vital Capacity; FEV1, Forced Expiratory Volume in 1 second; PEF, peak expiratory flow; PIF, peak inspiratory flow, MEF75, maximum expiratory flow at 75% of forced vital capacity; MMEF75/25, maximal mid-expiratory flow at 75-25% of forced vital capacity; FIV1, forced inspiratory volume in 1 second; FEF50/FIF50, ratio of forced expiratory flow at 50% of forced vital capacity to forced inspiratory flow at 50% of forced vital capacity. HR, heart rate; SBP, systolic blood pressure; DBP, diastolic blood pressure; MAP, mean arterial pressure; CO, Cardiac Output; CI, cardiac index; SV, stroke volume; SI, stroke index; SVR, systemic vascular resistance; SVRI, systemic vascular resistance index; TFC, thoracic fluid content; TFCI, thoracic fluid content index; VI, velocity index; ACI, acceleration index; PEP, pre-ejection period; LVET, left ventricular ejection time; STR, systolic time ratio; BMI, body mass index.**


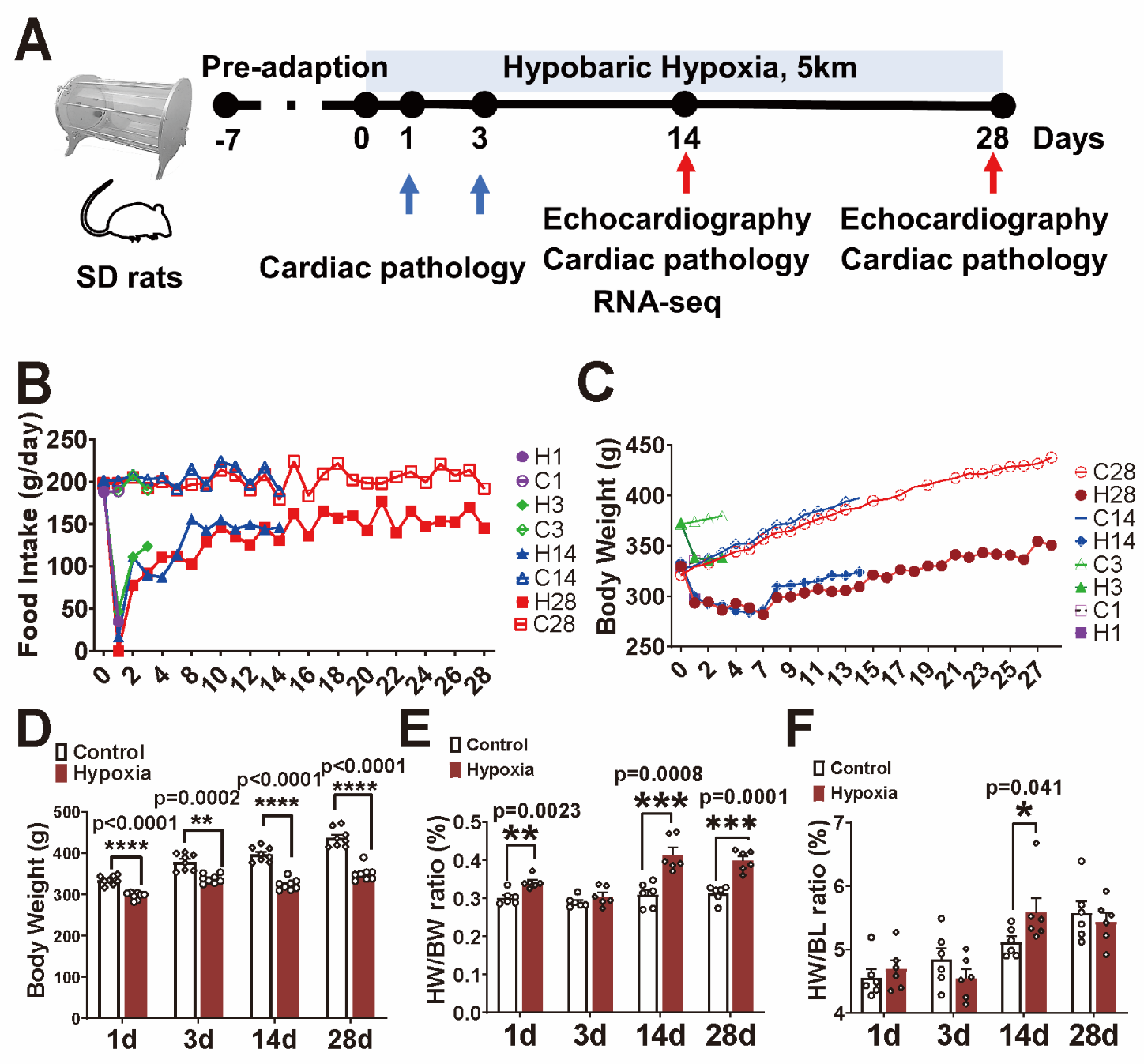


**Figure S2.** **Hypoxia simulated 5000m induced weight loss and heart hypotrophy. (A)** Schematic representation of the experimental design. (**B, C)** Dynamic alteration of food intake and body weight during hypoxia or normoxia treatment of each group. N=8. (**D)** Body weight. (**E)** Ratio of heart wet weight to body weight (HW/BW). (**F**) Ratio of heart wet weight to body length (nasoanal length). N=6.


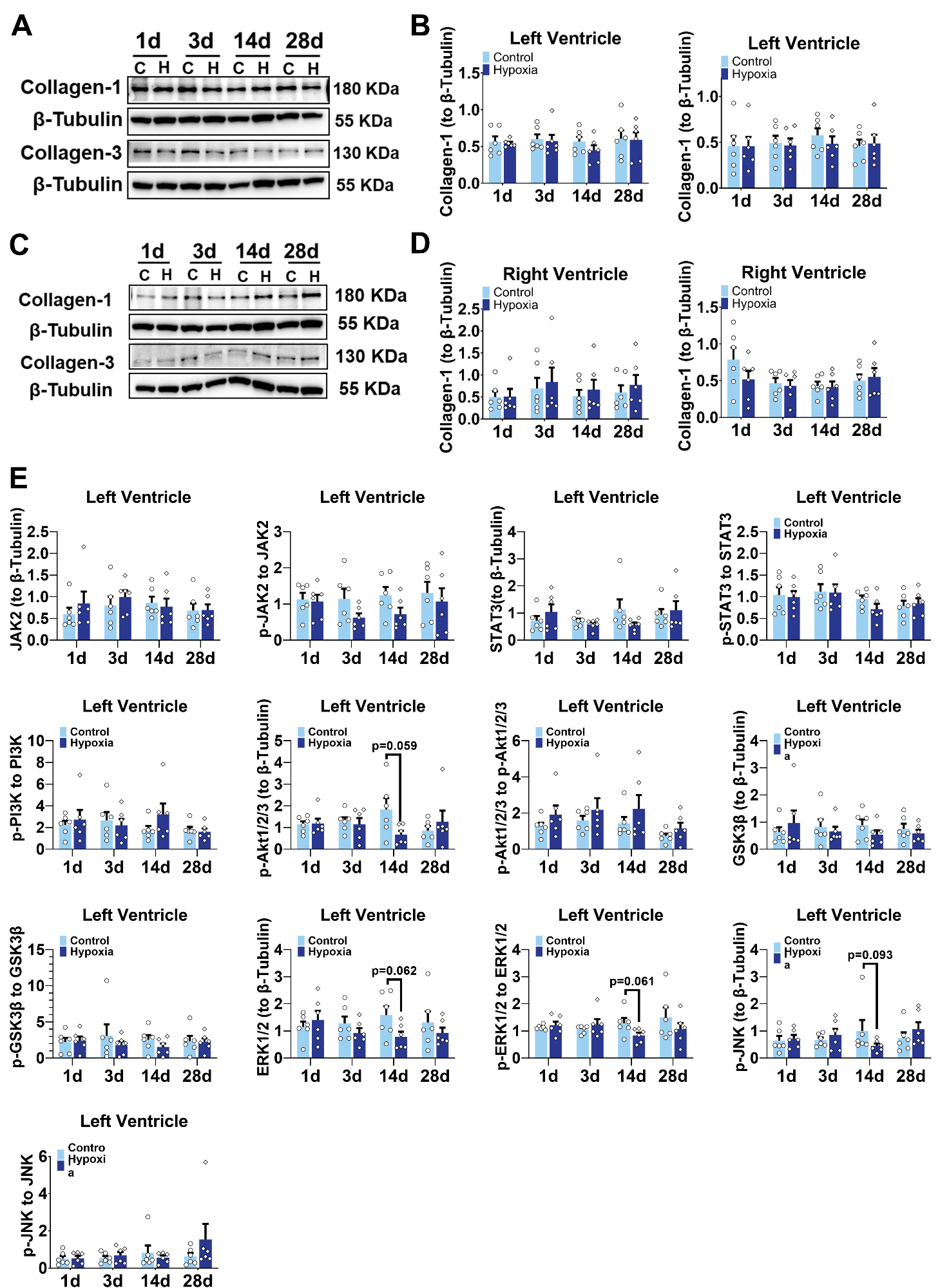


**Figure S3. Simulated exposure to hypoxia at 5000m induced alteration of fibrosis, and leptin signaling pathways.** (A) Western blot analysis and (B) Qualification of Collagen I and Collagen III in the left ventricle of SD rats. (C) Western blot analysis and (D) Qualification of Collagen I and Collagen III in the right ventricle of SD rats. (E) Western blot analysis of JAK2/STAT3, PI3K/Akt/GSK3β, ERK/JNK total protein and phosphorylated protein in left ventricles. n=6 per group. Results are presented as mean ± SEM.


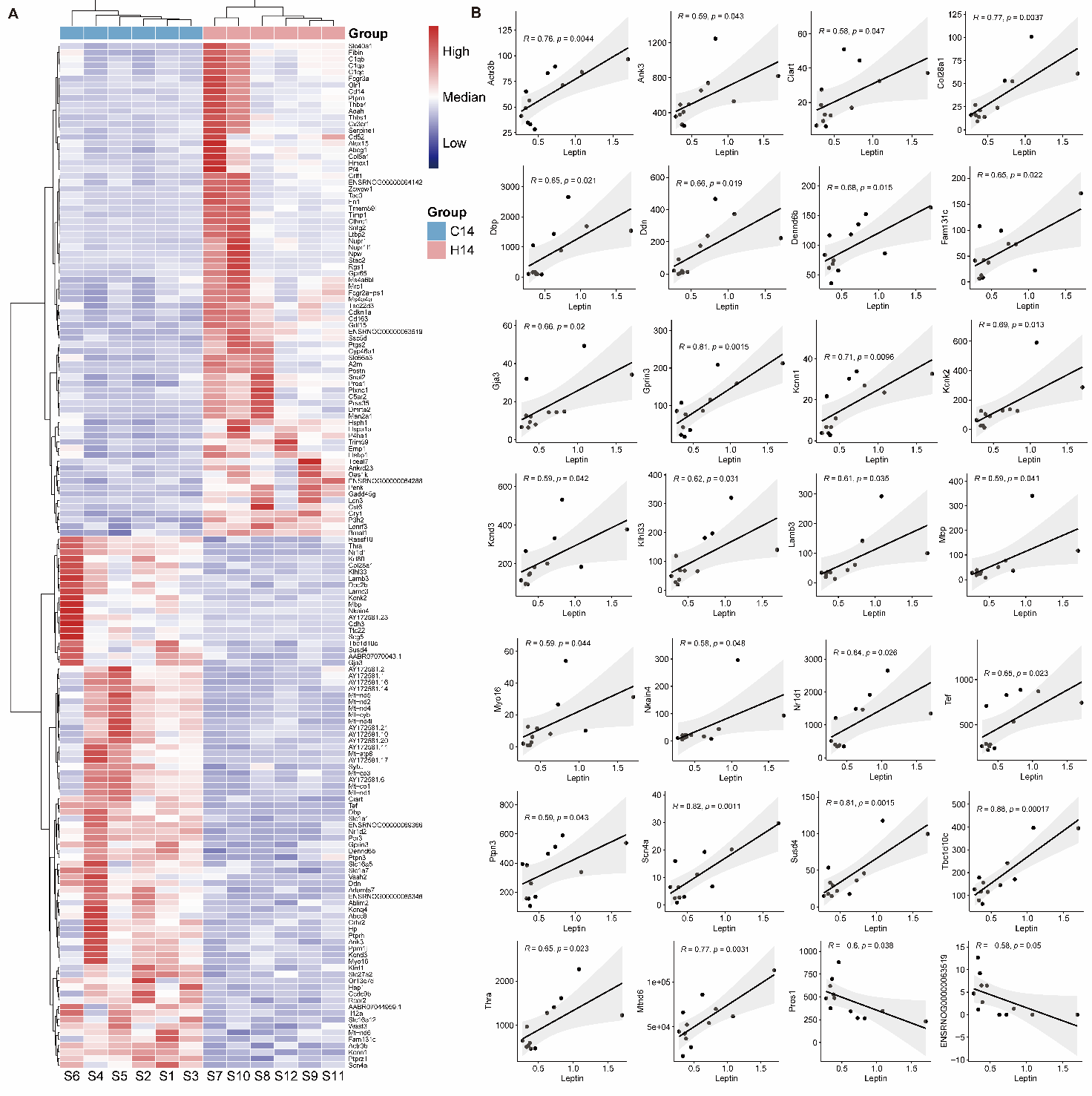


**Figure S4. Transcriptomic analysis of left ventricular myocardial tissues in rats and the association with leptin.** (A) Heatmap of DEGs (B) Correlation of Leptin with DEGs.


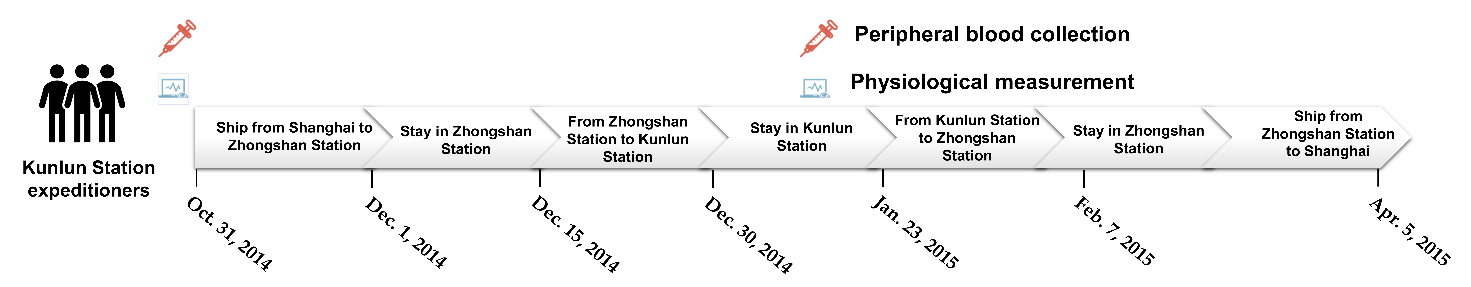


**Figure S5.** **Schedule of Chinese Antarctic Kunlun Station expedition.** On October 31, 2014, the expedition team departed on the Xuelong ship from Shanghai. After a 30-day voyage, they arrived at Zhongshan Station (69°22´S, 76°22´E, 10 m) on December 1, 2016. After 15 days of rest and preparation, they set off for Kunlun on December 15, traveling by snow vehicle for 15 days and arriving at Dome A Kunlun Station (80°22´S, 77°22´E, 4087 m) on December 30, 2014. At Kunlun Station, participants conducted scientific investigations for 24 days. On January 23, 2015, they evacuated from Kunlun Station and began their return journey after a brief stay at Zhongshan Station starting on February 7, 2015, and arrived in Shanghai on April 5. Peripheral blood samples were collected at two time points during the expedition, along with physiological examinations on October 30, 2014, and a 20-day stay at Kunlun Station on January 18, 2015.

**References:**

1 Toczylowski, K. *et al.* Plasma concentration and expression of adipokines in epicardial and subcutaneous adipose tissue are associated with impaired left ventricular filling pattern. *Journal of translational medicine* **17**, 310, doi:10.1186/s12967-019-2060-7 (2019).

2 Wang, P. *et al.* Pericardial Adipose Tissue-Derived Leptin Promotes Myocardial Apoptosis in High-Fat Diet-Induced Obese Rats Through Janus Kinase 2/Reactive Oxygen Species/Na+/K+-ATPase Signaling Pathway. *Journal of the American Heart Association* **10**, e021369, doi:10.1161/jaha.121.021369 (2021).

3 Barouch, L. A., Berkowitz, D. E., Harrison, R. W., O'Donnell, C. P. & Hare, J. M. Disruption of leptin signaling contributes to cardiac hypertrophy independently of body weight in mice. *Circulation* **108**, 754-759, doi:10.1161/01.Cir.0000083716.82622.Fd (2003).

4 McGaffin, K. R., Zou, B., McTiernan, C. F. & O'Donnell, C. P. Leptin attenuates cardiac apoptosis after chronic ischaemic injury. *Cardiovascular research* **83**, 313-324, doi:10.1093/cvr/cvp071 (2009).

5 McGaffin, K. R. *et al.* Leptin signalling reduces the severity of cardiac dysfunction and remodelling after chronic ischaemic injury. *Cardiovascular research* **77**, 54-63, doi:10.1093/cvr/cvm023 (2008).

6 Hall, M. E., Maready, M. W., Hall, J. E. & Stec, D. E. Rescue of cardiac leptin receptors in db/db mice prevents myocardial triglyceride accumulation. *Am J Physiol Endocrinol Metab* **307**, E316-325, doi:10.1152/ajpendo.00005.2014 (2014).

7 Abe, Y. *et al.* Leptin induces elongation of cardiac myocytes and causes eccentric left ventricular dilatation with compensation. *American journal of physiology. Heart and circulatory physiology* **292**, H2387-2396, doi:10.1152/ajpheart.00579.2006 (2007).

8 Gómez-Hurtado, N. *et al.* Beneficial effects of leptin treatment in a setting of cardiac dysfunction induced by transverse aortic constriction in mouse. *The Journal of physiology* **595**, 4227-4243, doi:<https://doi.org/10.1113/JP274030> (2017).

9 Takatani-Nakase, T. & Takahashi, K. Leptin suppresses non-apoptotic cell death in ischemic rat cardiomyocytes by reduction of iPLA(2) activity. *Biochemical and biophysical research communications* **463**, 13-17, doi:10.1016/j.bbrc.2015.05.008 (2015).

10 Abd Alkhaleq, H. *et al.* Leptin modulates gene expression in the heart and cardiomyocytes towards mitigating ischemia-induced damage. *Experimental cell research* **397**, 112373, doi:10.1016/j.yexcr.2020.112373 (2020).
