# Supplementary figures and images for: Association of attenuated leptin signaling pathways with impaired cardiac function under prolonged high-altitude hypoxia
Source: Sci Rep. 2024 May 3;14:10206. doi: 10.1038/s41598-024-59559-6 (PMC11068766; doi:10.1038/s41598-024-59559-6)

Left ventricle

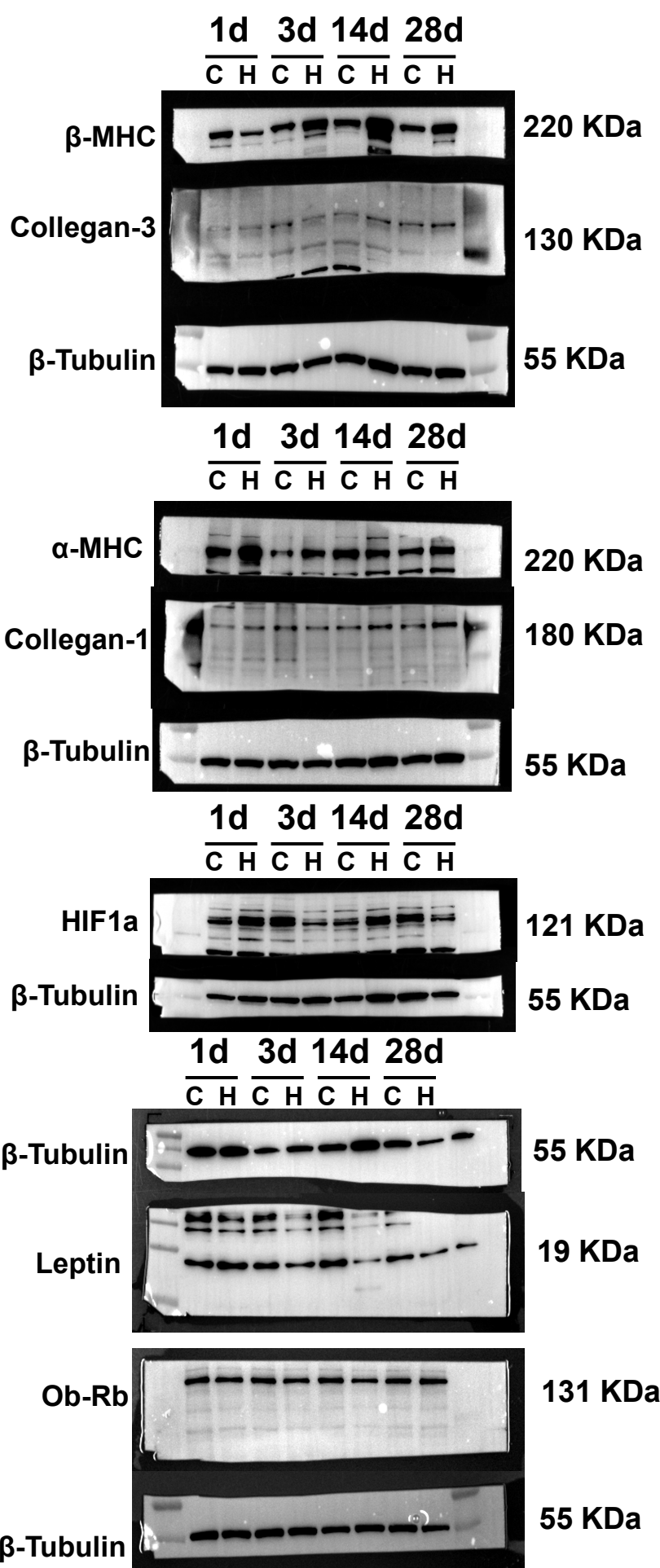

Right ventricle

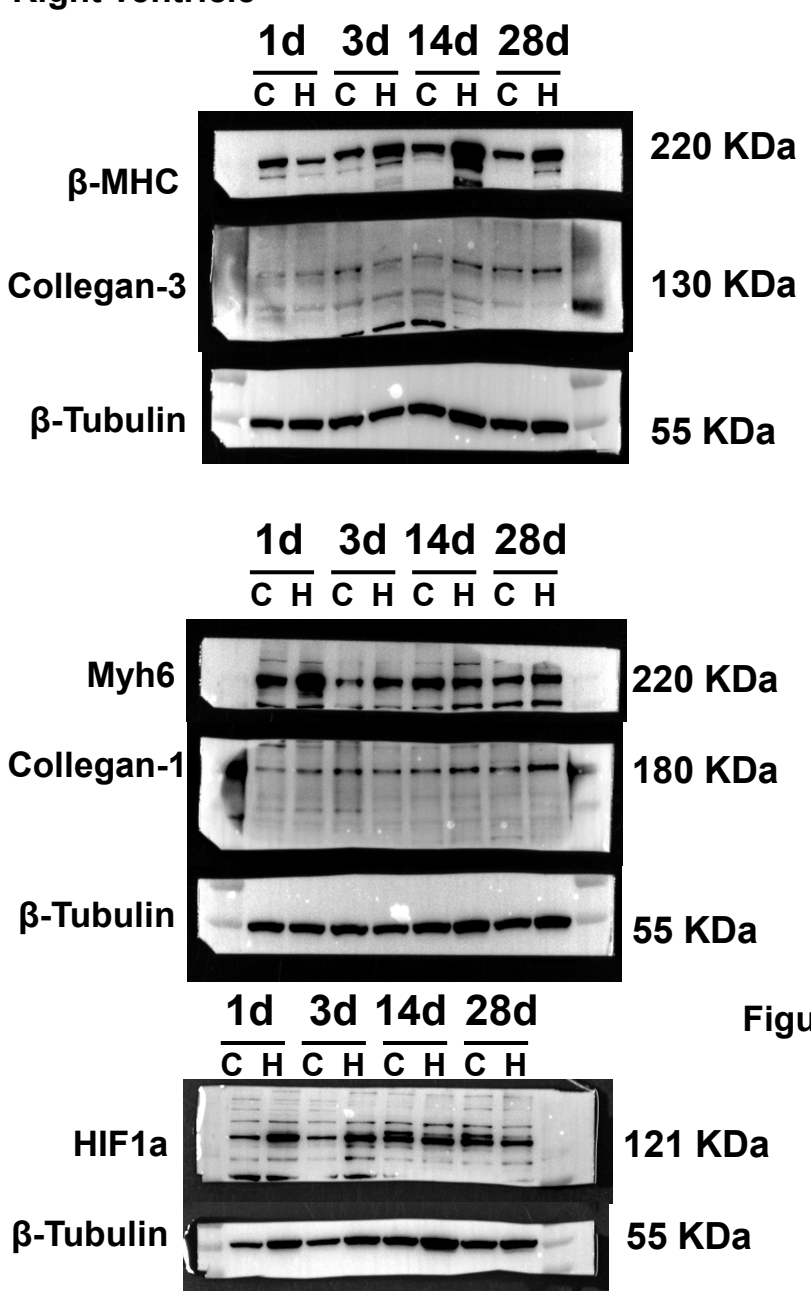

Figure 5

Right ventricle

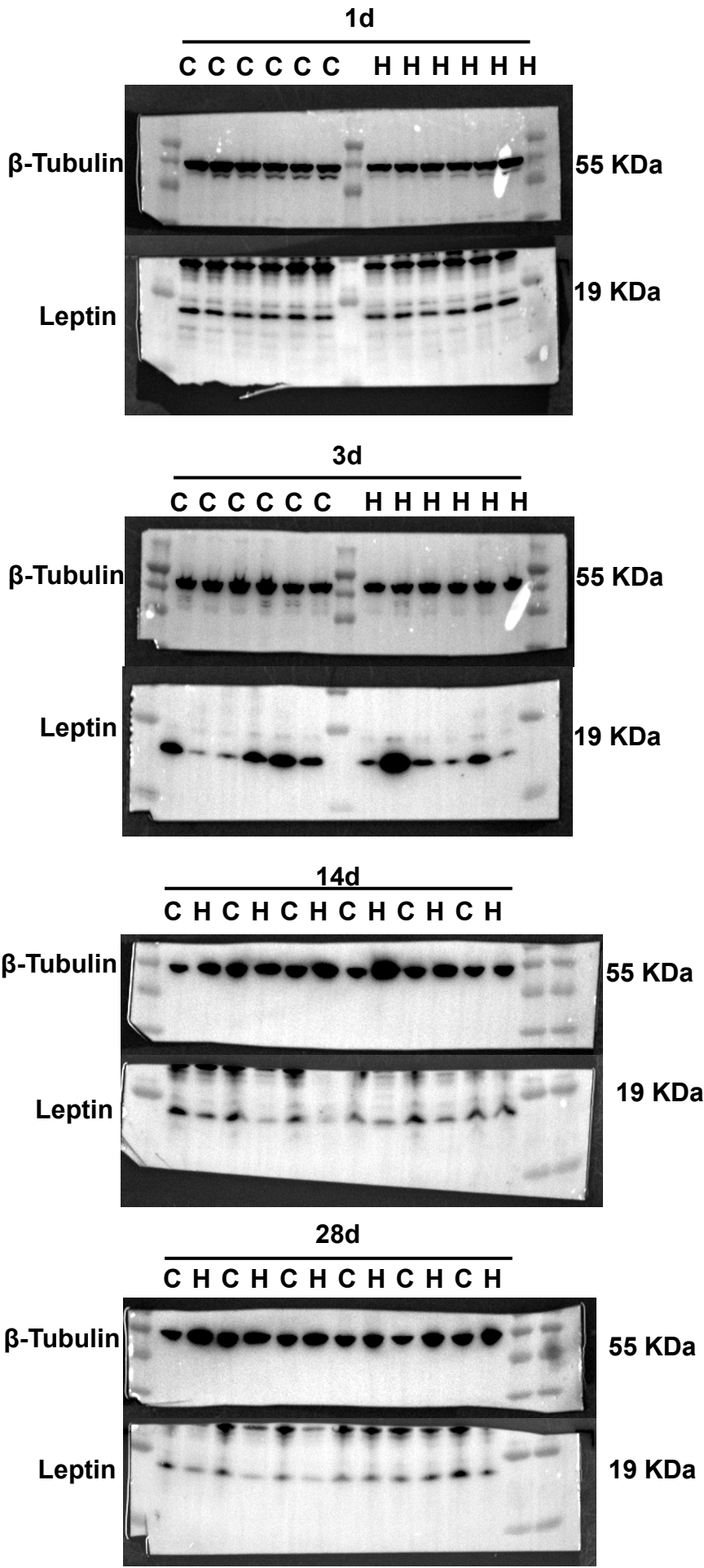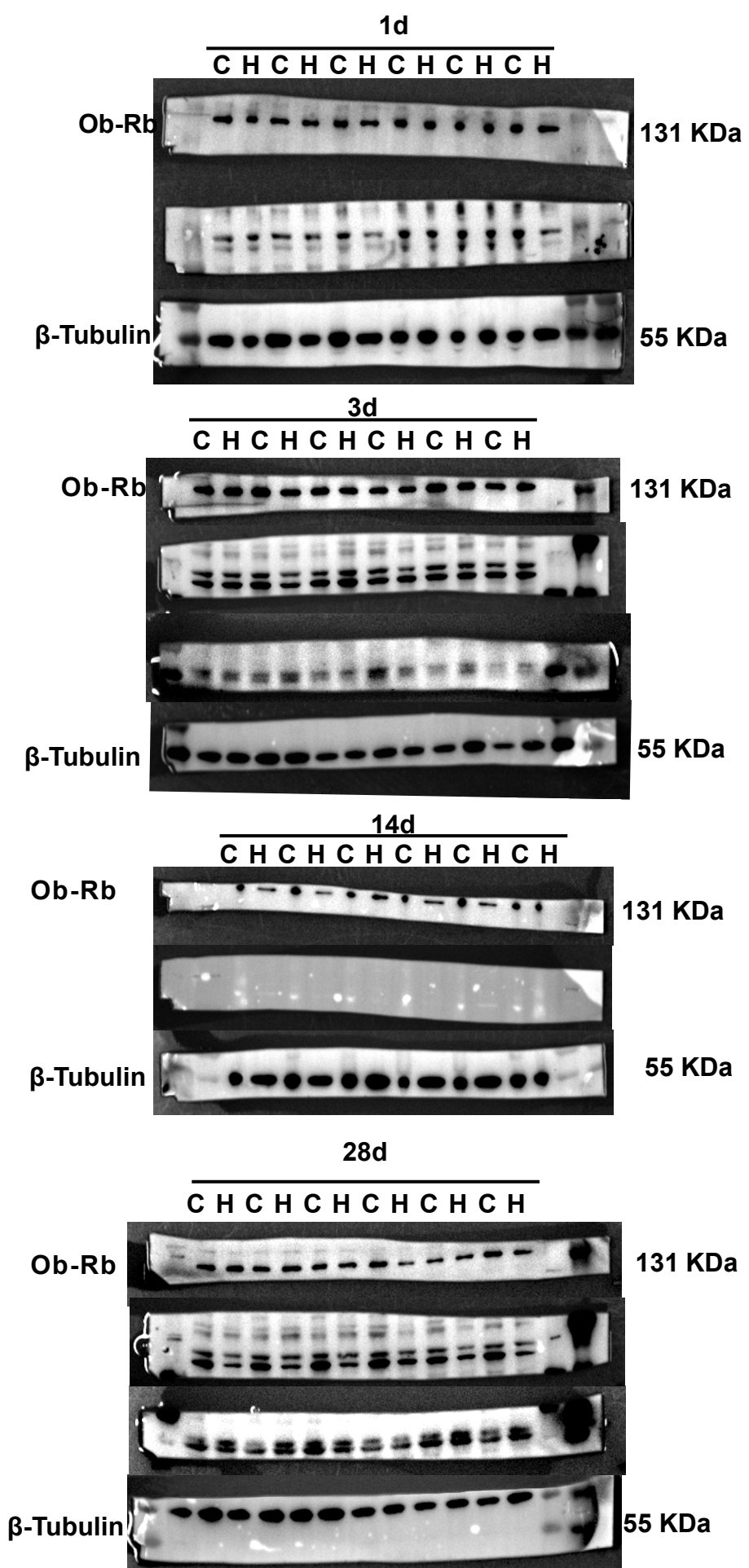

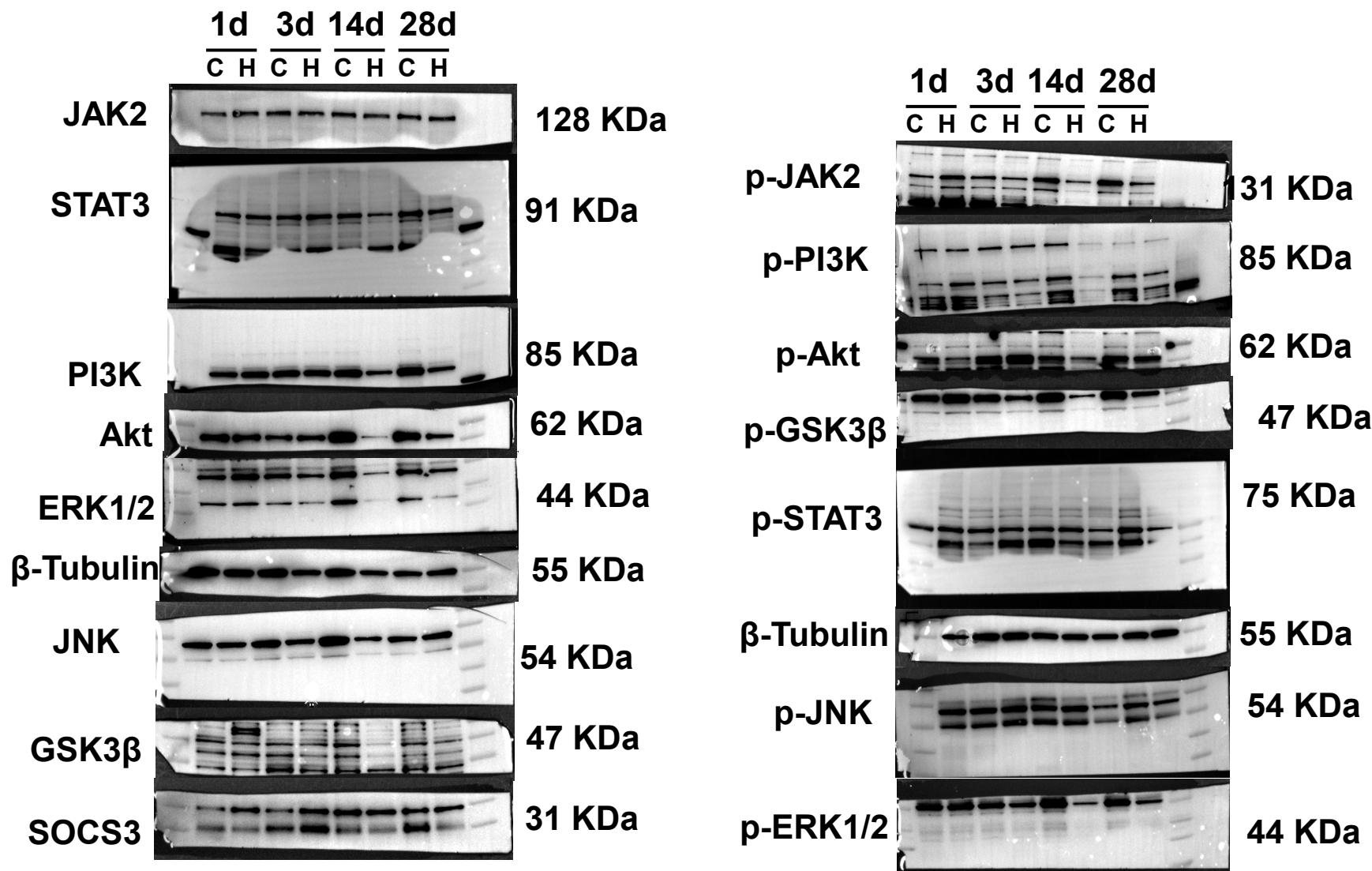

Supplement: Supplementary file 2 — Supplementary Figures. [file 41598_2024_59559_MOESM2_ESM.pdf]
